# Genetic mapping using a wheat multi-founder population reveals a locus on chromosome 2A controlling resistance to both leaf and glume blotch caused by the necrotrophic fungal pathogen *Parastagonospora nodorum*

**DOI:** 10.1007/s00122-019-03507-w

**Published:** 2020-01-29

**Authors:** Min Lin, Beatrice Corsi, Andrea Ficke, Kar-Chun Tan, James Cockram, Morten Lillemo

**Affiliations:** 1 Department of Plant Sciences, Norwegian University of Life Sciences, Post Box 5003, 1432 Ås, Norway; 2 John Bingham Laboratory, NIAB, Huntingdon Road, Cambridge, CB3 0LE UK; 3 Norwegian Institute of Bioeconomy Research, Høgskoleveien 7, 1433 Ås, Norway; 4 Centre for Crop and Disease Management, School of Molecular and Life Sciences, Curtin University, Bentley, WA Australia

## Abstract

**Key message:**

A locus on wheat chromosome 2A was found to control field resistance to both leaf and glume blotch caused by the necrotrophic fungal pathogen *Parastagonospora nodorum*.

**Abstract:**

The necrotrophic fungal pathogen *Parastagonospora nodorum* is the causal agent of *Septoria nodorum* leaf blotch and glume blotch, which are common wheat (*Triticum aestivum* L.) diseases in humid and temperate areas. Susceptibility to *Septoria nodorum* leaf blotch can partly be explained by sensitivity to corresponding *P. nodorum* necrotrophic effectors (NEs). Susceptibility to glume blotch is also quantitative; however, the underlying genetics have not been studied in detail. Here, we genetically map resistance/susceptibility loci to leaf and glume blotch using an eight-founder wheat multiparent advanced generation intercross population. The population was assessed in six field trials across two sites and 4 years. Seedling infiltration and inoculation assays using three *P. nodorum* isolates were also carried out, in order to compare quantitative trait loci (QTL) identified under controlled conditions with those identified in the field. Three significant field resistance QTL were identified on chromosomes 2A and 6A, while four significant seedling resistance QTL were detected on chromosomes 2D, 5B and 7D. Among these, *QSnb.niab*-*2A.3* for field resistance to both leaf blotch and glume blotch was detected in Norway and the UK. Colocation with a QTL for seedling reactions against culture filtrate from a Norwegian *P. nodorum* isolate indicated the QTL could be caused by a novel NE sensitivity. The consistency of this QTL for leaf blotch at the seedling and adult plant stages and culture filtrate infiltration was confirmed by haplotype analysis. However, opposite effects for the leaf blotch and glume blotch reactions suggest that different genetic mechanisms may be involved.

**Electronic supplementary material:**

The online version of this article (10.1007/s00122-019-03507-w) contains supplementary material, which is available to authorized users.

## Introduction

*Septoria nodorum* blotch (SNB), caused by the necrotrophic pathogen *Parastagonospora* (synonyms *Septoria, Stagonospora*) *nodorum* (Berk.) is one of the most important fungal diseases of wheat (*Triticum aestivum* L.) and has been reported in almost all wheat-producing areas worldwide (Ficke et al. 2018; Francki 2013; Friesen et al. 2007; Oliver et al. 2012). It can cause lesions on both wheat leaves and glumes, and can reduce grain yield by 30% (Bhathal et al. 2003; Wicki et al. 1999). Infected seeds and wheat debris are the primary inoculum sources, with infection favored by warm and humid conditions at later wheat developmental stages, as the asexual pycnidiospores are dispersed by rain-splash (Blixt et al. 2008; King et al. 1983; Ruud and Lillemo 2018; Sommerhalder et al. 2011). Currently, control of SNB relies heavily on fungicide application. Due to its mixed reproduction system, the genetic diversity and evolutionary potential of the pathogen population is considerable (McDonald and Linde 2002; Stukenbrock et al. 2006). Therefore, regardless of the environmental side effects caused by fungicide application, the risk of losing the chemical control efficacy is quite high as pathogen populations are being exposed to high selection pressure against limited groups of fungicides (Pereira et al. 2017). Thus, improving wheat genetic resistance to SNB is both a more environmentally friendly and durable method to control SNB. However, SNB resistance is controlled by many genes with additive effects (Friesen and Faris 2010). The durability of cultivar resistance to SNB is also challenged by the variability of the pathogen population within and between locations.

As a necrotrophic pathogen, the host interaction of *P. nodorum* follows an inverse gene-for-gene model (Friesen et al. 2007) whereby necrotrophic effectors (NEs) produced by the pathogen interact with corresponding host sensitivity loci (*Snn*) and trigger programmed cell death in host tissues. By definition, the necrotrophic pathogen feeds on dying tissues and benefits from the host-NE interactions to expand infection. *P. nodorum* NEs are small secreted proteins, previously called host-selective toxins (HSTs), which act as virulence factors facilitating disease development (Liu et al. 2004a, b). Up to now, eight NEs have been identified which interact with nine wheat sensitivity loci (Ruud and Lillemo 2018; Shi et al. 2015). Among those, only three *P. nodorum* NE genes have been cloned: *ToxA*, *Tox1* and *Tox3* (Friesen et al. 2009; Liu et al. 2009, 2012). In addition, two of the host sensitivity genes have been cloned in wheat: *Tsn1* and *Snn1* (Faris et al. 2010; Shi et al. 2016). Interestingly, both genes encode receptor-like proteins, classes of genes which are well known for controlling disease resistance to biotrophic pathogens. For example, *Tsn1* encodes a protein containing a nucleotide-binding site and leucine-rich repeats (NBS-LRR) (Faris et al. 2010), while *Snn1* encodes a wall-associated kinase (WAK). Based on these results, Shi et al. (2016) hypothesized that necrotrophic pathogens hijack the signaling pathways of plant resistance to biotrophs and manipulate it to become a susceptibility pathway for necrotrophs.

In addition, NE-*Snn* interactions have been reported to underlie the molecular basis of the quantitative susceptibility for SNB leaf blotch (Friesen and Faris 2010). NE-*Snn* interactions were first identified under greenhouse conditions using plants at the seedling stage, where Tox1 was characterized as a host-selective toxin (HST) which interacted with the *Snn1* locus on the short arm of chromosome 1B (Liu et al. 2004a, b). Since then, additional NE-*Snn* interactions have been found to be relevant to field SNB resistance/susceptibility. For example, Friesen et al. (2009) evaluated the BR34 × Grandin wheat mapping population in the field using artificial *P. nodorum* inoculation, finding *Tsn1* and *Snn2* to confer susceptibility under field conditions. Via inoculation with *P. nodorum* isolate SN15, Phan et al. (2016) found the Tox1-*Snn1* interaction as contributing to SNB susceptibility at both the seedling and adult plant stage. Similarly, a recent study by Ruud et al. (2017) confirmed a major effect of *Snn3*-*B1* in field susceptibility, finding this locus to be significant in 2 years out of a four-year field study. In contrast to the established relevance of sensitive NE-*Snn* interactions to field resistance, correlations between seedling resistances and adult plant resistances are low (Shankar et al. 2008). This might be because isolates used in such seedling tests produce different NEs in comparison with the NEs which showed effects in the field (Ruud and Lillemo 2018). For instance, Ruud et al. (2017) found that when *P. nodorum* isolate 201618 which lacks the *Tox3* gene was used for seedling testing, correlation between seedling disease scores and field disease scores was less significant than those for Tox3 producing isolates. Further isolation of NEs, surveys of NE genes and alleles in current *P. nodorum* isolates collected from the field and additional studies of seedling and field QTL resistance in different host genetic backgrounds are needed to provide a clearer picture of the pathways and genes that control SNB resistance/susceptibility.

While both leaf blotch and glume blotch are caused by the same pathogen on the same host, the inheritance of resistance to glume blotch is reported to be genetically different from leaf blotch (Chu et al. 2010; Wicki et al. 1999; Xu et al. 2004). Eighteen QTL have previously been identified for glume blotch resistance on chromosomes 2A, 2B, 2D, 3B, 4B, 5A, 6B and 7D, reviewed by Ruud and Lillemo (2018). However, most glume blotch studies were undertaken before Friesen et al. (2007) hypothesized the inverse gene-for-gene model for leaf blotch, and the resistance/sensitivity mechanism for glume blotch is still unclear (Solomon et al. 2006; Uphaus et al. 2007; Wainshilbaum and Lipps 1991; Wicki et al. 1999). Linkage mapping with bi-parental populations has widely been used for detecting and localizing genes for quantitative traits such as SNB (Friesen et al. 2007; Ruud et al. 2017). As only two alleles segregate at a given QTL in such populations, the power of QTL detection is generally high, and therefore, high genetic map resolution is usually not required (Cavanagh et al. 2008; Cockram and Mackay 2018; Kover et al. 2009). However, low recombination rates in standard bi-parental populations derived from a single round of intercrossing limit QTL mapping resolution for a given population size, potentially making them less amenable for fine mapping (Bandillo et al. 2013; Cavanagh et al. 2008; Huang et al. 2012). One alternative approach to linkage mapping is using collections of unrelated lines for genome wide association scans (GWAS), which is efficient especially for collections with low linkage disequilibrium (LD) (Gupta et al. 2014; Korte and Farlow 2013; Pascual et al. 2016). The genetic variability for the target trait is usually much higher in an association mapping (AM) panel, as multiple alleles may exist per locus and high genetic recombination rates are captured due to the historic recombination within the genealogy of the panel (Gupta et al. 2014; Mackay et al. 2009). However, GWAS in AM panels also has its own specific limitations. For example, genetic subpopulation structure should be taken into account; otherwise, it will result in high risk of false positive associations (Breseghello and Sorrells 2006; Gupta et al. 2014; Sneller et al. 2009). Multiparent advanced generation intercross (MAGIC) population designs include higher allelic diversity and higher genetic recombination rate than equivalently sized bi-parental populations and avoid the loss of power resulting from correction for subpopulation structure in AM panels (Cavanagh et al. 2008; Mackay et al. 2014). As a result, MAGIC populations can be used for both coarse mapping and fine mapping at relatively high resolution (Cavanagh et al. 2008; Stadlmeier et al. 2019). The recently developed wheat eight-founder ‘NIAB Elite MAGIC’ population (Mackay et al. 2014) is estimated to capture around 80% of the single nucleotide polymorphism (SNP) variation in north-western European wheat germplasm (Gardner et al. 2016) and includes founders of prominence within the European wheat pedigree (Fradgley et al. 2019). In addition, this population has been used to fine map the *Snn1* and *Snn3*-*B1* effector sensitivity loci (Cockram et al. 2015; Downie et al. 2018). Therefore, the population is well suited to survey the occurrence of *P. nodorum* resistance loci within a multi-site, multi-year experimental design.

Here, we used the ‘NIAB Elite MAGIC’ population to (1) identify QTL associated with leaf blotch sensitivity or resistance by both seedling and field testing, (2) investigate the relationship between effector/seedling sensitivity and field SNB resistance, (3) compare QTL identified for leaf blotch from different experimental locations and (4) compare QTL identified for both leaf blotch and glume blotch to investigate the relationship between the host resistance/sensitivity mechanism against these diseases.

## Materials and methods

### Plant material

The ‘NIAB Elite MAGIC’ population has been previously described (Mackay et al. (2014). The founders (Alchemy, Brompton, Claire, Hereward, Rialto, Robigus, Soissons and Xi19) are elite winter wheat cultivars selected to capture key traits, such as high yield and good disease resistance. Briefly, the population was derived by intercrossing the eight founders over three generations, followed by multiple rounds of selfing to produce homozygous recombinant inbred lines (RILs). The full set of the population consists of more than 1000 RILs. In this study, a subset of 486 lines were tested in Norway both in the greenhouse for seedling resistance/susceptibility to leaf blotch and in the field for both leaf blotch and glume blotch resistance/susceptibility in adult plants. In the UK, 498 lines were tested for leaf blotch resistance/susceptibility in the field.

### Field trials

In total, six autumn sown field trials were undertaken across two locations (four in Norway and two in the UK). In the 2014 field season, leaf blotch field trials were conducted with a subset 187 MAGIC RILs and seven of the founders (Alchemy, Brompton, Claire, Hereward, Robigus, Soissons and Xi19) at the Vollebekk Research Station in Ås, Norway. From 2016 to 2018, a subset of 486 RILs and all eight founders were tested in hillplot (small plots sown 50 cm apart in rows, 40 cm between rows) trials at the Vollebekk research station. Naturally *P. nodorum* infected straw was put out in the field as inoculum early in the season before stem elongation. Plots were arrayed using an incomplete alpha lattice design, with founders and additional controls being repeated ten times. Mist irrigation for 5 min every half hour from 10 am to 8 pm was undertaken to promote SNB infection. From 2016 to 2018, the selective fungicide Forbel 750 (Bayer Crop Science, a.i.: Phenpropimorph) was applied (750 g/ha Phenpropimorph) every 3 weeks from stem elongation to the end of the disease scoring to control stripe rust and powdery mildew. This fungicide has little to no effect on *P. nodorum* infection.

In the UK, two field trials were conducted (2017 and 2018), at NIAB, Cambridge, UK. The trial consisted of 498 RILs in two reps each, and eight founders in four reps each, plus 29 additional controls in four or five reps each, considered interesting for some characteristics. The trials consisted of 1178 plots, with each plot consisting of two 1 m rows. The agronomy packages used are listed in Supplementary Table S11. Trial design was undertaken in R (R Core Team 2015) using the package Blocks Design v2.8, and each trial arranged in two randomized, complete replicates, each of 13 blocks. Mist irrigation was applied for 20 min twice a day. The same fungicide program described above was applied. Representative UK *P. nodorum* isolates were used to inoculate the UK field trials. A spore suspension (5 × 10^6^ spores/mL) was used to inoculate the trials with sprayers. The inoculation was carried out once a week for 2 weeks, once the plants reached growth stage 39 (GS39, flag leaf fully visible).

### Field phenotypic evaluation

#### Leaf blotch

Leaf blotch severity in Norway was scored four times in the 2014 trial, three times in each of the 2016 and 2017 season trials, and twice in 2018 (due to hot and dry weather). The first scoring was done when the most susceptible line reached 70% severity (approximately the ‘early dough’ stage, GS83), and then, the second and third scoring were each undertaken approximately 1 week after the previous scoring. Disease severity was estimated visually as the percentage of leaf area with leaf blotch symptoms in each hillplot canopy. In the UK, a 0-to-9 qualitative lesion-type rating was used to evaluate each variety. A score of 0 = the absence of visible lesions; 2 = 1 lesion per 10 tillers; 3 = 2 small lesions per tiller; 4 = small lesions beginning to form areas of dead tissue across the width of the leaf; 5 = large areas of diseased tissue covering 1/3 of the leaf surface; 6 = infected tissue covering half of the leaf surface; 7 = infected tissue covering most of the leaf, more than the green tissue remaining; 8 = very little green tissue left on the leaf; 9 = large coalescent lesions with no green tissue remaining. The first score was undertaken when the 5% of the total plots showed symptoms of the disease and then once a week. Disease severity was scored a total of four times in 2017 and five times in 2018.

### Glume blotch

Glume blotch was scored in the same field trials as leaf blotch in Norway, but only once per season, in 2016 and 2017. The date of scoring was immediately after the final leaf blotch scoring. The glume blotch scoring system was based on the percentage of infected glume area in each hillplot canopy. As naturally infected straw was used as inoculum, it took time for the disease to advance from the lower leaves to the spikes. Glume blotch was not scored in the 2018 season owing to insufficient disease development due to the dry and warm weather and the resulting early maturity.

### Other traits

Plant height was measured as the height from ground to either the bottom of the spikes (Norway) or to the top of the spike (UK). Heading date was scored in both countries when the majority of plants within a plot had fully emerged ears.

### Seedling inoculation experiments and *P. nodorum* isolates

Three *P. nodorum* isolates were used in the seedling study. Accessions 203667 and 203649 were Norwegian single spore isolates collected from wheat leaf samples. Isolate 203667 was collected from the winter wheat cultivar Olivin at Staur, Hedmark, Norway, in 2015. Isolate 203649 was collected from the winter wheat cultivar Kuban at Sarpsborg, Østfold, Norway, in 2015. Isolate 202579 is a Mexican isolate collected from Tlanepantla, Estado de Mexico, Mexico, in 2007, and is commonly used for SNB inoculation at the International Maize and Wheat Improvement Center (CIMMYT) (CIMMYT accession: CIMFU 463). Isolates were grown on Potato Dextrose Agar (PDA) for 2 weeks in darkness at temperature around 20 °C in order to obtain enough mycelium for DNA extraction. The DNEasy Plant Kit (Qiagen) was used for DNA extraction following the manufacturer’s instructions. Genotyping of the three necrotrophic effector genes *ToxA*, *Tox1* and *Tox3* was undertaken as described by Gao et al. (2015).

*P. nodorum* isolates were grown for 7 days on V8-PDA media in an incubation chamber with 24 h white and near ultraviolet light (NUV) at around 20 °C to enhance sporulation. Pycnidiospores were used to prepare spore suspension, and the final concentration of the spore suspension was adjusted to 1 × 10^6^ spores/mL for inoculation. Tween 20 was added to the spore suspension to reduce surface tension at a concentration of one drop per 50 mL.

Three to four seeds of each of the 472 MAGIC RILs and the 8 founders were sown in plastic cones fitting a 98 cone-rack (Stuewe and sons, Tangent, Orlando, USA) filled with peat soil (Gartnerjord, Tjerbo, Norway). Entries were randomly assigned across 8 blocks (60 entries per block) using an incomplete block design. The SNB susceptible cultivar Brakar was sown as border plants to reduce edge effect. Prior to inoculation, seedlings were grown in a greenhouse at a temperature of 20/16 °C (day/night), 65% humidity and 16 h light cycle for 14 days. Inoculation was undertaken by spraying the spore suspension onto 14-day-old plants until runoff. Inoculated plants were first placed in a mist chamber with 100% relative humidity for 24 h and then returned to the greenhouse. The second leaf of each plant was scored for disease severity using a 0–5 scale, where 0 indicated highly resistant and 5 indicated highly susceptible to SNB (Liu et al. 2004b), 7 days post-inoculation. Each experiment was repeated three times.

### ToxA production

Heterologous expression of ToxA was undertaken in *Escherichia coli* BL21E using the pET21a expression vector, as previously described (Tan et al. 2012), undertaken at the Protein Expression Facility (The University of Queensland). ToxA preparations were desalted in 20 mM sodium phosphate pH 7.0 m freeze-dried for storage, and re-suspended prior to use in ultra-pure water and stored at 4 °C.

### Seedling infiltration using culture filtrates and ToxA

Three to four seeds of each MAGIC line were sown in plastic cones following the protocol listed above for the inoculation experiments. *P. nodorum* isolates were cultivated in liquid Fries 3 medium (Friesen and Faris 2012) for the production of necrotrophic effectors. Three weeks after the stationary phase, culture filtrates were sterilized filtered through membranes filters (white gridded: 0.45 μm, diameter: 47 mm, S-PAK, France) and roughly 50 μL culture filtrates or ToxA preparation were infiltrated into the second leaf of each plant by using a 1-mL syringe with the needle removed. The reactions to isolate 203649 and 202579 were scored 5 days post-infiltration using a 0–4 scale (Tan et al. 2012), where score 0 indicates no symptoms, 1 indicates slight chlorosis, 2 indicates extensive chlorosis, 3 indicates complete chlorosis without tissue collapse, and 4 indicated complete necrosis. The reaction to isolate 203667 and ToxA were scored using a 0–3 scale (Friesen and Faris 2012), where 0 indicates no symptoms, 1 is mottled chlorosis, 2 is complete chlorosis without tissue collapse, and 3 is complete necrosis. Individual seedlings of each genotype growing in the same cone were used as replicates.

### Statistical analysis

For leaf blotch and glume blotch phenotypic data, the average scores from the three to four timepoints measured for each trait were calculated for each line and then corrected for block effects using SAS v.9.4 (SAS Institute Inc.) to estimate the mean disease severity of each line and variances. For the straw-inoculated field trials in Norway, plant height and days to heading were used as covariates in multi-linear regression to calculate corrected disease severities. This was done using R Studio version 1.1.442 (RStudio Team 2015) by subtracting the estimated disease severities based on the fitted model from the observed field severities recorded in the field. For leaf blotch data from the spray-inoculated trials in the UK, neither plant height nor heading date were significantly correlated with disease scores. Therefore, the mean disease severities were used without correction of confounding traits. Since few variations were explained by the first scoring in 2018 of the UK trial, average disease scores were calculated by taking the average of the second to fifth scores.

The calculations of the Pearson correlation coefficients were carried out in R Studio using the package Hmisc (Harrell 2019). Paired Wilcoxon signed-rank test was carried out using R Studio. Broad sense heritability of line means was calculated as broad sense by first estimating components of variation from REML while taking into account all features of the experimental designs. Heritability was then estimated as *h*^2^ = *σ*^2^*G*/(*σ*^2^*G* + *σ*^2^*e*) where *σ*^2^*G* is the genetic variation between line means and *σ*^2^*e* is the error variance appropriate to those means. Calculations were carried out in GenStat (VSN International 2011) and the package lme4 (Bates et al. 2015) in R Studio.

### QTL mapping

The 643 NIAB Elite MAGIC RILs were previously genotyped at the F_5_ generation using the 90 K SNP array (Wang et al. 2014) resulting in 20,643 polymorphic SNPs (Mackay et al. 2014; Gardner et al. 2016), and the data used to make a genetic map consisting of 18,601 SNPs (Gardner et al. 2016). Of these, markers assigned to the 7367 unique map positions were used for QTL mapping. QTL analyses were carried out using haplotype analyses, using the 7369 SNPs that map to unique positions in the MAGIC genetic map (Gardner et al. 2016). Founder haplotype probabilities were calculated using the ‘mpprob’ function in R/mpMap (Huang and George 2011) implemented in R/qtl (Broman et al. 2003) with a threshold of 0.5. QTL analysis using these haplotype probabilities was carried out via two methods: (a) by linear mixed model using all mapped markers (termed here ‘identity by descent’ mapping, IBD), and (b) by interval mapping using the ‘mpIM’ mapping function in R/mpMap, with the inclusion of 0 (interval mapping, IM), 5, or 10 covariates (composite interval mapping, CIM). For IBD analysis, correction for multiple testing was accounted for by using a significance threshold of *q* = 0.05 using the package R/qvalue. For interval mapping, two significance thresholds were used: (1) using the ‘sim.sigthr’ function from R/mpMap package, 100 simulations of the dataset were conducted based on no QTL hypothesis, followed by calculation of the genome wide p value, and determination of the significance threshold using *p* = 0.05. QTL above this permutated significance threshold are designated here as ‘strong QTL.’ (2) An arbitrary threshold of − log_10_(*p*) = 3. QTL with − log_10_(*p*) between 3 and the permutated threshold or QTL explaining > 5% of phenotypic variation but − log_10_(*p*) lower than 3 are designated here as ‘weak QTL.’ A full QTL model was then fitted with all QTL using R/fit.mpQTL. IM was used to call QTL, with additional detection using CIM-cov5, CIM-cov10 and IBD used to further confirm IM QTL calls. Significance values and percentage variation explained for all QTL reported in the manuscript are derived from IM. Flanking markers were defined by CIM-cov10 when QTL were detected by both IM and CIM; otherwise, intervals were defined by IM.

DNA sequences flanking selected SNP markers within QTL intervals were obtained from the website https://triticeaetoolbox.org, allowing SNPs to be anchored to the wheat cv. Chinese Spring reference genome assembly (IWGSC RefSeq v1.0; IWGSC et al. 2018) via BLASTn analysis.

### Haplotype analysis

Haplotype analysis was performed for the QTL *QSnb.niab*-*2A.3*. Two peak markers (*BS00062679_51* and *RAC875_c9372_94*) from *QSnb.niab*-*2A.3* were selected for constructing haplotypes. The mean corrected disease severities for the population were calculated based on haplotypes. Kruskal–Wallis test was calculated using the R/pgirmess package (Giraudoux 2018) in R Studio, and the significant interval was obtained by *p* < 0.05.

## Results

### Phenotypic evaluation of field resistance

**Fig. 1:**
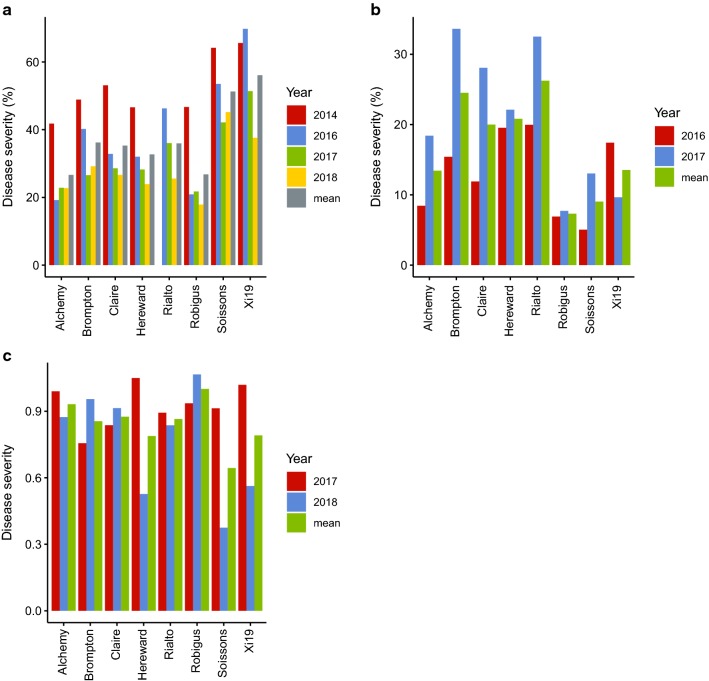
Disease severity of the MAGIC founders in different years and locations. Mean disease severity of each line is indicated. **a** Leaf blotch severity in Ås, Norway, **b** glume blotch severity in Ås, Norway, **c** leaf blotch severity in Cambridge, UK

The eight MAGIC founders showed different levels of SNB severity in all 4 years in Norway, except Rialto which was not tested in 2014 (Fig. 1a, b). Alchemy and Robigus were relatively resistant to leaf blotch, as low levels of infection were observed in all years, while Soissons and Xi19 were more susceptible compared to the other parents (Fig. 1a). However, the disease severity of the founders in the UK trials did not show the same trend of severity as observed in Norway (Fig. 1c). For glume blotch, Brompton and Rialto were the most susceptible, while Alchemy and Robigus were relatively resistant (Fig. 1b).

**Fig. 2:**
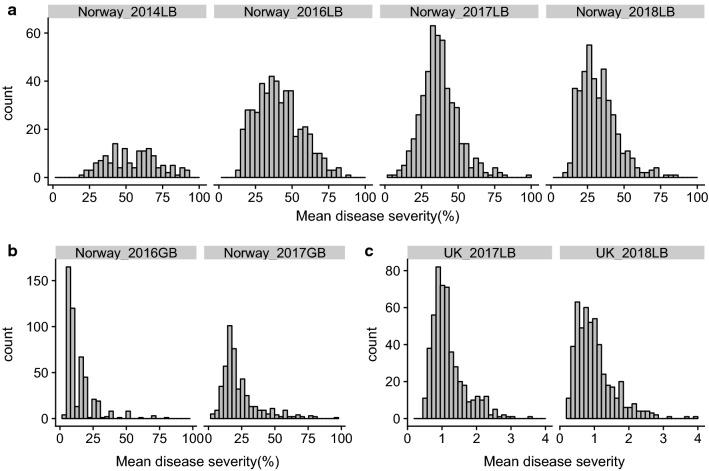
Disease severity of the MAGIC population in different years and locations. **a** Leaf blotch severity in Ås, Norway, **b** glume blotch severity in Ås, Norway, **c** leaf blotch severity in Cambridge, UK, *LB* leaf blotch, *GB* glume blotch

Broad variation in leaf blotch severity among the MAGIC RILs indicated that the inheritance of SNB resistance was quantitative (Fig. 2a). For glume blotch, the majority of lines over all tested years varied between 0 and 25% infection (Fig. 2b). The range of leaf blotch disease severity was from 0 to 100% in all 4 years in Norway (2014, 2016–2018). Due to dry and hot conditions, only 425 lines yielded reliable data that were included for QTL analysis in 2018, and the overall infection level was lower compared to 2016 and 2017 (Fig. 2a).

Significant negative correlation between leaf blotch severity (LB) and plant height (PH) was observed in all tested years in Norway except 2014 (Table 1). The correlation coefficients in year 2016, 2017 and 2018 were − 0.22, − 0.22 and − 0.21, respectively (*p* < 0.0001). Similarly, the correlation between days to heading (DH) and LB was also significant in each year: − 0.30 (*p* < 0.0001) in 2014, − 0.30 (*p* < 0.0001) in 2016, − 0.22 (*p* < 0.0001) in 2017, while slightly less significant in 2018 (*r* = −0.10, *p* < 0.05). There was also significant negative correlation between glume blotch severity and PH in all years in Norway (Table 1). However, DH was positively correlated with glume blotch: 0.21 (*p* < 0.0001) in 2016 and 0.10 (*p* < 0.05) in 2017. Neither PH nor DH was significantly correlated with leaf blotch in the UK (Table 1).

**Table 1:** Pearson correlation coefficients for leaf blotch (LB) and glume blotch (GB) severities, days to heading (DH) and plant height (PH) within years

	2016NLB	2016NGB		2017NLB	2017NGB		2018NLB
16DH	− 0.30***	0.21***	17DH	− 0.22***	0.10*	18DH	− 0.10*
16PH	− 0.22***	− 0.34***	17PH	− 0.22***	− 0.40***	18PH	− 0.21***

	2017ULB		2018ULB		2014NLB
17DH	− 0.04	18DH	0.05	14DH	− 0.30***
17PH	− 0.03	18PH	− 0.04	14PH	− 0.07

Trials are coded to indicate year (2016, 2017 and 2018), country (*N* Norway, *U* UK) and disease (*LB* leaf blotch, *GB* glume blotch)

***< 0.0001; *< 0.05

After correction for the effects of PH and DH in the Norway trials, leaf blotch severities were all significantly correlated between years and locations, except for LB in the UK 2017 and LB in Norway 2014 (Table 2). Similarly, the correlation of corrected glume blotch severity between 2016 and 2017 was also significant (*r* = 0.31, *p* < 0.0001). For the same year same location, correlations between leaf blotch and glume blotch were also significant: *r* = 0.22 (*p* < 0.0001) in 2016 and 0.13 in 2017 (*p* < 0.01) (Table 2). Heritability (h^2^) for leaf blotch in Norway was between 48.00 and 77.45% among years, while in the UK heritability was 13.61% and 57.25% in 2017 and 2018, respectively (see Supplementary Table S1).

**Table 2:** Pearson correlation coefficients for leaf blotch and glume blotch severities between years, after correction for the effects of plant height and days to heading

	2014NLB	2016NLB	2016NGB	2017NLB	2017NGB	2018NLB	2017ULB
2016NLB	0.36***						
2016NGB	0.07	0.22***					
2017NLB	0.27**	0.50***	0.04				
2017NGB	− 0.04	0.06	0.31***	0.13**			
2018NLB	0.23**	0.29***	− 0.01	0.36***	− 0.13*		
2017ULB	0.09	0.24***	0.00	0.21***	0.00	0.22***	
2018ULB	0.18*	0.17**	0.04	0.17**	0.07	0.11*	0.23***

Trials are coded to indicate year (2016, 2017 and 2018), country (*N* Norway, *U* UK) and disease (*LB* leaf blotch, *GB* glume blotch)

***< 0.0001; **< 0.01; *< 0.05

### Genetic analysis of field experiments

**Fig. 3:**
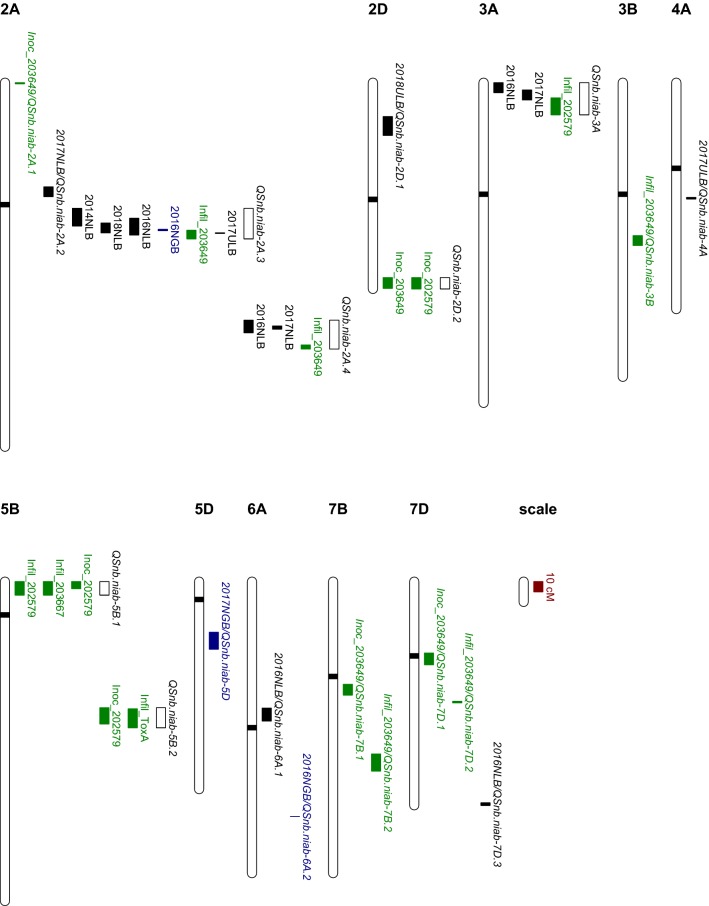
Genetic map locations of all QTL detected in this study. QTL locations and interval sizes are indicated by bars on the right hand side of each chromosome and are based on the genetic marker information in Table 3. Field leaf blotch QTL are indicated in black, field glume blotch QTL in blue (*N* Norway, *U* UK, *LB* leaf blotch, *GB* glume blotch), and seedling QTL in green (Inoc: greenhouse inoculation, Infil: greenhouse infiltration). Of these QTL, those detected in more than one environment are indicated using a white bar, along with the designated QTL name assigned in this study

**Fig. 4:**
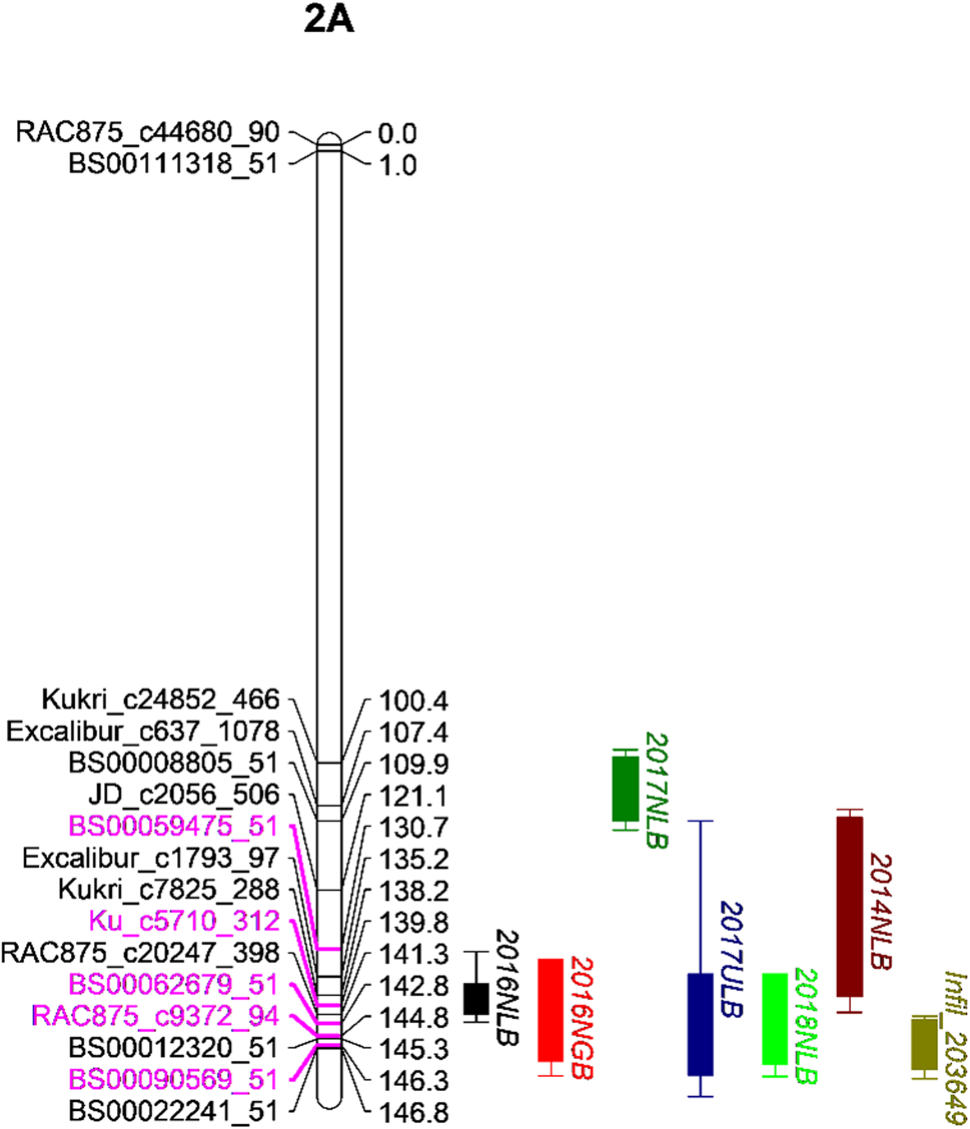
Genetic map of the *QSnb.niab*-*2A.3* locus and on the short arm of chromosome 2A in the NIAB Elite MAGIC population. *N* Norway, *U* UK, *LB* leaf blotch, *GB* glume blotch, Infil: infiltration, peak markers are indicated in pink

**Fig. 5:**
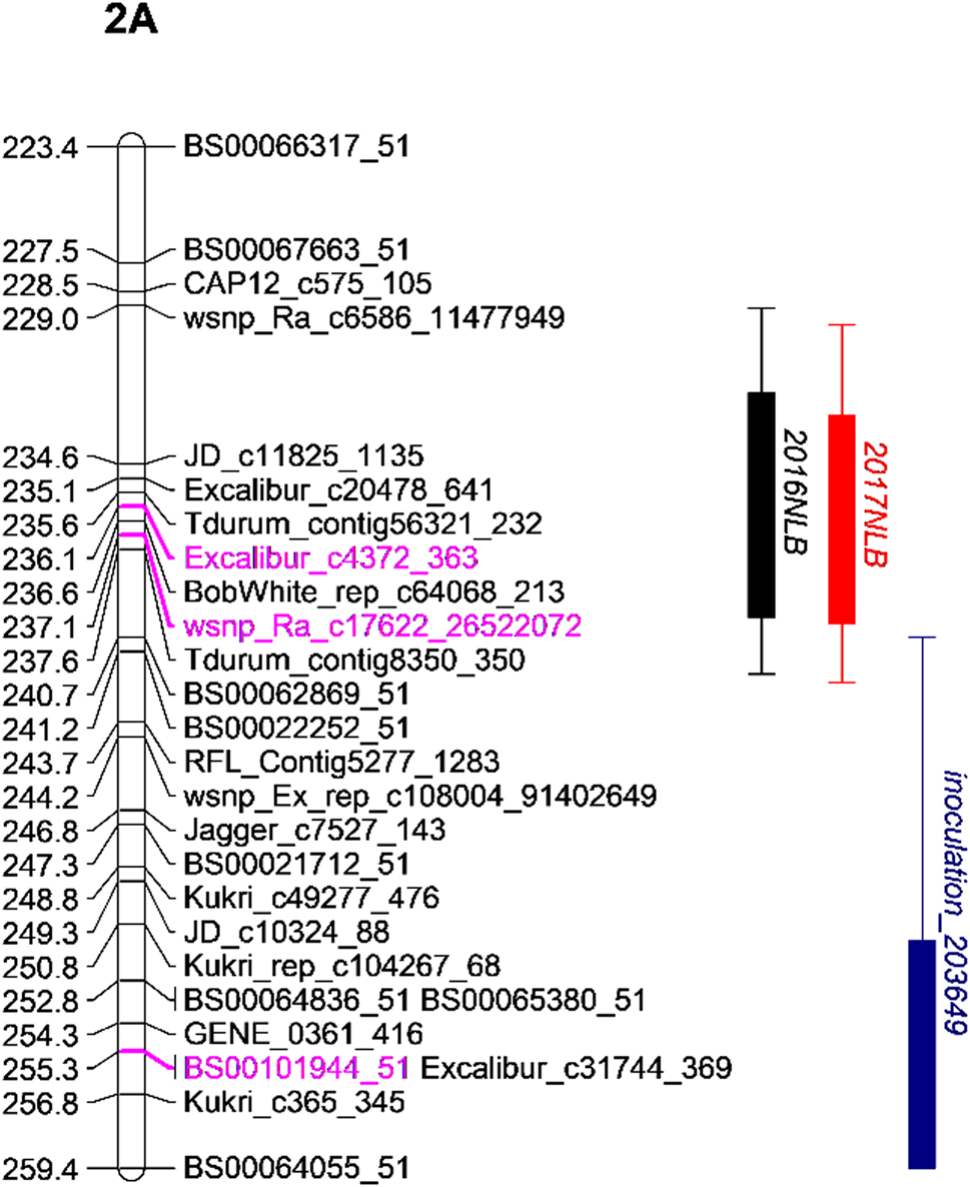
Genetic map of the *QSnb.niab*-*2A.4* locus on the long arm of chromosome 2A in the NIAB Elite MAGIC population. *N* Norway, *LB* leaf blotch. Peak markers are indicated in pink

Sixteen QTL (− log_10_(*p*) > 3) were identified by IM/CIM using field data for leaf blotch from six trials across two locations and glume blotch for 2 years at one location (Table 3; Fig. 3). Among them, 10 QTL were detected for leaf blotch in Norway, three QTL were detected for leaf blotch in the UK, and three QTL were detected for glume blotch in Norway. QTL were mapped to chromosomes 2A, 3A, 4A, 5D, 6A and 7D (Table 3). As some QTL were located to overlapping chromosomal regions and were significant in multiple years and/or environments, these were subsequently grouped into ten distinct genetic loci. Of these, three were above the permutated *p* = 0.05 significance threshold (Table 3): (1) QTL *QSnb.niab*-*2A.3* on the short arm of chromosome 2A was detected as a ‘strong’ QTL for leaf blotch in Norway during 2014, 2016 and 2018 and glume blotch in Norway in 2016, explaining 16%, 6.8%, 6.57% and 4.12% of the phenotypic variation, respectively (Table 3; Fig. 4). *QSnb.niab*-*2A.3* was additionally detected as a ‘weak’ QTL (− log_10_(*p*) = 3.17) in the 2017 UK trial, explaining 3.87% of the phenotypic variation. Anchoring the most significant SNP markers to the wheat genome assembly found *QSnb.niab*-*2A.3* to be approximately located at 574–635 Mb on chromosome 2A. (2) The ‘strong’ QTL *QSnb.niab*-*2A.4* on the long arm of chromosome 2A was identified in Norway 2016, and as a ‘weak’ QTL in Norway 2017, explaining 3.74% and 4.53% of the phenotypic variation, respectively. The *QSnb.niab*-*2A.4* peak marker was located at 237.13 cM in 2016 (SNP *wsnp_Ra_c17622_26522072*, − log_10_(*p*) = 4.41, 759 Mb) and at 236.12 cM in 2017 (SNP *Excalibur_c4372_363*, − log_10_(*p*) = 3.97, 758 Mb) in 2017 (Table 3; Fig. 5). (3) *QSnb.niab*-*6A.1*, identified as a ‘strong’ QTL for leaf blotch resistance in Norway 2016, was located at 129 cM (SNP *TA004558_1018*, 97.81 Mb) and explained 3.85% of the phenotypic variation. Genetic analysis of plant height, flowering time and SNB for Norway trials unadjusted for the effect of plant height and days to heading is presented in Supplementary Tables S6-S9 and discussed in more detail in Supplementary Text 1. In summary, the confounding effects of plant height and days to heading influenced the detection of glume blotch-related QTL more than leaf blotch, as all strong QTL detected by unadjusted glume blotch phenotypic data co-located with plant height QTL. Except one ‘weak QTL’ *QDh.niab*-*6A* on chromosome 6A detected for days to heading in Norway in 2014 might co-locate with adjusted leaf blotch QTL *QSnb.niab*-*6A.1* detected in Norway in 2016. No other plant height or days to heading QTL were found to co-locate with both adjusted and unadjusted leaf blotch QTL. However, in general for both leaf blotch and glume blotch, less QTL were detected by unadjusted data and QTL detected using adjusted data were found to be less significant when using unadjusted data.

**Table 3:** *P. nodorum* resistance/sensitivity QTL identified in the ‘NIAB Elite MAGIC’ population from field trials, seedling culture filtrate infiltration, ToxA infiltration and seedling inoculation

QTL	Ref.	Trait	Year	Chr	Interval (cM)	Flanking markers	Peak marker	−log_10_ (*p*)	IWGSC RefSeq v1.0 start (bp)	IWGSC RefSeq v1.0 end (bp)	R^2^ (%)	Detected by QTL methods
*QSnb.niab*-*2A.1*	1	Inoc_203649		2A	0–1.01	RAC875_c44680_90 and BS00111318_51	BS00111318_51	3.00	2378473	2378574	5.11	IM,CIM (cov5, cov10),IBD
*QSnb.niab*-*2A.2*		LB (Norway)	2017	2A	100.36–109.94	Kukri_c24852_466 and BS00008805_51	Excalibur_c637_1078	3.19	78844360	111457371	3.88	IM,CIM (cov5, cov10)
***QSnb.niab*-*2A.3***		**LB** (Norway)	**2014**	**2A**	**121.07–138.24**	**JD_c2056_506 and Kukri_c7825_288**	**BS00059475_51**	**5.82**	**410872779**	**588258104**	**16.00**	IM,CIM (cov5, cov10),IBD
***QSnb.niab*-*2A.3***		**LB** (Norway)	**2018**	**2A**	**135.19–146.82**	**Excalibur_c1793_97 and BS00022241_51**	**RAC875_c9372_94**	**4.67**	**558953000**	**663329017**	**6.80**	IM,CIM (cov5, cov10),IBD
***QSnb.niab*-*2A.3***		**LB** (Norway)	**2016**	**2A**	**130.65–146.82**	**BS00059475_51 and BS00022241_51**	**Ku_c5710_312**	**6.20**	**574172945**	**663329017**	**6.57**	IM,CIM (cov5, cov10),IBD
***QSnb.niab*-*2A.3***		**GB** (Norway)	**2016**	**2A**	**141.26–142.77**	**RAC875_c20247_398 and BS00062679_51**	**BS00062679_51**	**4.21**	**611946675**	**615287757**	**4.12**	IM,CIM (cov5, cov10),IBD
*QSnb.niab*-*2A.3*		Infil_203649 (ncov1)^†^		2A	142.2–150.6	BS00022641_51 and IAAV4015	BS00090569_51	3.49	612422267	677529836	2.98	CIM (cov1, cov5, cov10)
*QSnb.niab*-*2A.3*		LB (UK)	2017	2A	144.81–145.31	RAC875_c9372_94 and BS00012320_51	RAC875_c9372_94	3.17	635606922	647927416	3.87	IM,CIM (cov5, cov10)
***QSnb.niab*-*2A.4***		**LB (Norway)**	**2016**	**2A**	**229.02–241.18**	**wsnp_Ra_c6586_11477949 and BS00022252_51**	**wsnp_Ra_c17622_26522072**	**4.41**	**755929525**	**775619939**	**3.74**	IM,CIM (cov5, cov10),IBD
*QSnb.niab*-*2A.4*		LB (Norway)	2017	2A	234.62–237.63	JD_c11825_1135 and Tdurum_contig8350_350	Excalibur_c4372_363	3.97	758396871	780714672	4.53	IM,CIM (cov5, cov10)
*QSnb.niab*-*2A.4*		Inoc_203649		2A	252.80–256.82	BS00064836_51 and Kukri_c365_345	BS00101944_51	3.17	761248555	765269621	5.06	IM,IBD
*QSnb.niab*-*2D.1*	2	LB (UK)	2018	2D	32.58–50.83	wsnp_JD_rep_c63957_40798083 and BobWhite_c59161_181	BS00029208_51	3.51	14897896	27859806	3.91	IM,CIM (cov5, cov10),IBD
***QSnb.niab*-*2D.2***		**Inoc_203649**		**2D**	**188.01–198.36**	**RFL_Contig1128_620 and Kukri_c36328_419**	**Excalibur_c42413_442**	**4.61**	**635950166**	**637636412**	**11.42**	IM,CIM (cov5, cov10),IBD
*QSnb.niab*-*2D.2*		Inoc_202579		2D	188.01–198.86	RFL_Contig1128_620 and BS00010685_51	Ra_c19051_1446	4.33	635950166	638147754	4.86	IM,CIM (cov5, cov10),IBD
*QSnb.niab*-*3A*		LB (Norway)	2016	3A	0–9.55	RAC875_c46403_277 and Kukri_rep_c69028_347	Tdurum_contig83209_316	3.95	1031134	10300906	4.37	IM,CIM (cov5, cov10),IBD
*QSnb.niab*-*3A*		LB (Norway)	2017	3A	7.04–16.64	Kukri_rep_c89183_282 and BS00066230_51	wsnp_Ex_c6833_11782875	4.09	8685996	32325166	4.31	IM,CIM (cov5, cov10)
*QSnb.niab*-*3A*		Infil_202579		3A	14.63–31.02	TA003589_0518 and RAC875_c20134_535	BS00055211_51	3.49	8865639	14851061	5.09	IM,CIM (cov5, cov10),IBD
*QSnb.niab*-*3B*		Infil_203649		3B	147.39–156.96	BS00076872_51 and Excalibur_c5977_1409	BobWhite_c13099_755	3.47	566843633	578616694	4.69	IM,CIM (cov5, cov10),IBD
*QSnb.niab*-*4A*		LB (UK)	2017	4A	110.56–112.09	BS00072025_51 and IAAV6581	Kukri_c96129_147	3.68	598590361	605713556	3.56	IM,CIM (cov5, cov10)
***QSnb.niab*-*5B.1****(Snn3-B1)*	**3, 4**	**Infil_202579**		**5B**	**0–13.16**	**Tdurum_contig44048_276 and wsnp_Ex_c26252_35497729**	**Tdurum_contig44048_276**	**5.48**	**13428116**	**27830730**	**8.10**	IM,CIM (cov5, cov10),IBD
***QSnb.niab*-*5B.1****(Snn3-B1)*		**Infil_203667**		**5B**	**0–13.16**	**Tdurum_contig44048_276 and wsnp_Ex_c26252_35497729**	**BS00022336_51**	**8.90**	**13428116**	**27830730**	**10.40**	IM,CIM (cov5, cov10),IBD
*QSnb.niab*-*5B.1 (Snn3-B1)*		Inoc_202579		5B	0–6.88	Tdurum_contig44048_276 and Kukri_c60322_490	Excalibur_rep_c104354_205	3.59	13428116	19438610	4.84	IM,CIM (cov10), IBD
***QSnb.niab*-*5B.2****(Tsn1)*	**4, 5**	**Inoc_202579**		**5B**	**123.06–138.93**	**IACX7649 and wsnp_Ex_c6695_11577150**	**Kukri_c54078_114**	**7.93**	**539294940**	**547404553**	**8.54**	IM,CIM (cov5, cov10),IBD
***Tsn1***		**Infil_ToxA**		**5B**	**124.06–142.56**	**IACX11840 and Excalibur_c33675_201**	**Kukri_c54078_114**	**Inf**	**539935182**	**550847459**	**73.43**	IM,CIM (cov5, cov10),IBD
*QSnb.niab*-*5D*	6	GB (Norway)	2017	5D	49.43–66.08	BobWhite_c7263_337 and BS00063971_51	BS00110475_51	3.57	389934855	431201019	4.44	IM,CIM (cov10),IBD
***QSnb.niab*-*6A.1***		**LB (Norway)**	**2016**	**6A**	**123.39–135.99**	**IAAV5188 and RFL_Contig3088_949**	**TA004558_1018**	**4.85**	**74025753**	**249160705**	**3.85**	IM,CIM (cov5, cov10),IBD
*QSnb.niab*-*6A.2*	7, 8	GB (Norway)	2016	6A	229.10	BS00096240_51 and GENE_4028_152	GENE_4028_152	3.68	600395629	600406208	3.56	IM,CIM (cov5, cov10),IBD
*QSnb.niab*-*7B.1*		Inoc_203649		7B	100.24–110.66	BS00067599_51 and Excalibur_rep_c67475_1420	BS00067599_51	4.31	115244415	498523237	4.06	IM,CIM (cov5, cov10), IBD
*QSnb.niab*-*7B.2*		Infil_203649		7B	168.06–184.77	Kukri_c15912_860 and Excalibur_c50612_409	BS00077956_51	2.91	673961429	700551772	5.83	IM,CIM (cov5, cov10), IBD
***QSnb.niab*-*7D.1***		**Inoc_203649**		**7D**	**69.65–81.20**	**GENE_4292_204 and BS00049220_51**	**GENE_4292_204**	**9.90**	**174445641**	**458015017**	**7.89**	IM,CIM (cov5, cov10),IBD
*QSnb.niab*-*7D.2*		Infil_203649		7D	116.86–118.37	BS00023150_51 and BS00070188_51	BS00070188_51	3.03	554596126	559460390	4.13	IM,IBD
*QSnb.niab*-*7D.3*		LB (Norway)	2016	7D	215.78–218.30	RAC875_c10022_23 and JD_c2708_1512	JD_c2708_1512	3.49	629325776	724128709	4.06	IM,CIM (cov5, cov10),IBD

Significance values and proportion of the variance explained (*R*^2^) for all QTL reported are derived from IM analysis and the intervals determined via CIM-cov10, unless otherwise indicated. References of studies where QTL were found in similar positions: 1—Rybak et al. (2017), 2—Phan et al. (2016), 3—Liu et al. (2009), 4—Downie et al. (2018), 5—Faris et al. (2010), 6—Liu et al. (2015), 7—Gao et al. (2015), 8—Arseniuk et al. (2004). QTL with − log_10_(*p*) value > 3 are presented, with QTL above the permutated trait-specific significance threshold highlighted in bold. Reference (Ref.), Chromosome (Chr.), IM (interval mapping), CIM (composite interval mapping), IBD (identity by descent). The − log10(*p*) value for *Tsn1* is recorded as ‘Inf,’ as the *p* value was 0, resulting in an error when converted to the log_10_ scale

^†^Detected via CIM-cov1, -cov5 and -cov10

### Phenotypic evaluation of seedling inoculation and infiltration

The *ToxA*, *Tox1* and *Tox3* profiles for the three isolates used for seedling experiments were determined using previously published assays (Gao et al. 2015). Norwegian isolate 203649 was found to lack the *ToxA*, *Tox1* and *Tox3* genes, and Norwegian 203667 possessed *ToxA* and *Tox3*, while isolate 202579 from CIMMYT (CIMFU 463) possessed all three effector genes.

#### Infiltration

**Fig. 6:**
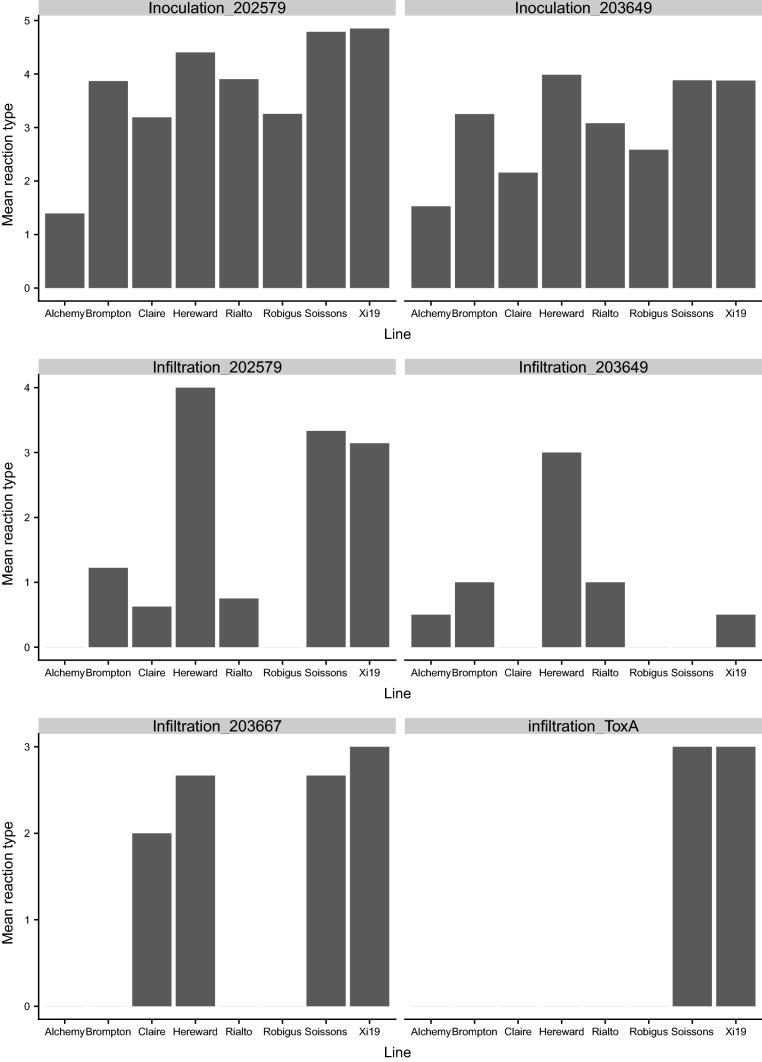
Reactions of MAGIC founders to infiltration (using isolate culture filtrate and the effector ToxA) and inoculation experiment treatment with two *P. nodorum* isolates. Isolate 203649 was found to lack the *ToxA*, *Tox1* and *Tox3* genes, 203667 possessed *ToxA* and *Tox3*, while isolate 202579 possessed all three effectors

**Fig. 7:**
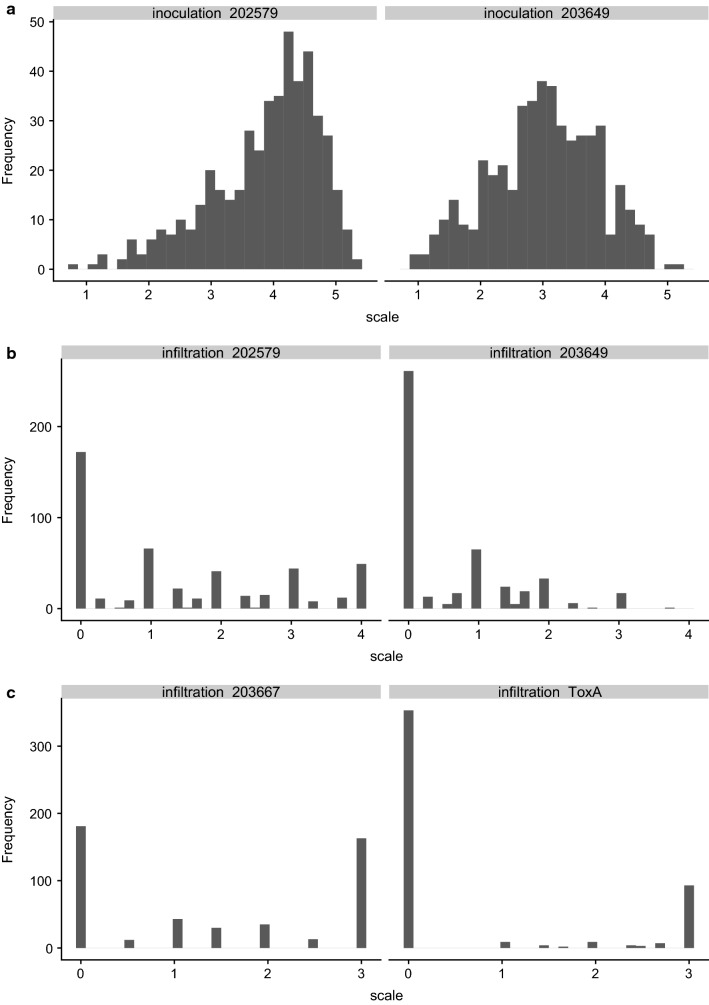
Seedling disease severity of the MAGIC population. **a** Inoculation with isolates 202579 and 203649, **b** infiltration with isolate 202579 and 203649, **c** infiltration with isolate 203667 culture filtrate and ToxA

The reactions of the eight MAGIC founders to *P. nodorum* infiltration (using culture filtrate or the effector ToxA) or inoculation (using spore suspensions) are shown in Fig. 6. Hereward was the most sensitive founder to culture filtrate from isolate 203649 (which does not produce any of the three toxins tested), while Claire, Robigus and Soissons showed a complete insensitive reaction. The remaining founders showed moderate susceptibility. However, very few MAGIC RILs had complete necrosis symptoms and even the most susceptible founder, Hereward, only had a reaction score of 3 (complete chlorosis without tissue collapse) using a 0–4 scoring scale. For infiltration with isolate 203667, Hereward, Soissons and Xi19 showed high sensitivity, and Claire was moderately sensitive, while the remaining founders were insensitive (Fig. 6). Infiltration with ToxA found Soissons and Xi19 to be sensitive (score = 3), while the rest of the founders were all insensitive (score = 0). 37.9% and 36.1% of the MAGIC RILs were insensitive to infiltration using culture filtrate from isolates 203667 and 202529, respectively, while 55.8% were insensitive to infiltration using isolate 203649. 34.1% of the MAGIC RILs were highly sensitive to infiltration using isolate 203667 culture filtrate (score = 3), and 19.2% were highly sensitive to infiltration using ToxA (score = 3), 10% were highly sensitive to infiltration using isolate 202579 (score = 4), while just one RIL was identified as possessing a sensitivity score of 4 to infiltration using culture filtrate from isolate 203649 (Fig. 7). Heritabilities (h^2^) for culture filtrate infiltration with isolate 203667, 203649, 202579 and infiltration with effector ToxA were 0.89, 0.84, 0.84 and 0.88, respectively.

#### Inoculation

Inoculation of the MAGIC founders using spore suspensions from each of the two isolates investigated (202759 and 203649) found the same trends in sensitivity as observed for culture filtrate infiltration, with Hereward, Soissons and Xi19 found to be the most susceptible, followed by Brompton and Rialto. Claire and Robigus were even less susceptible, while Alchemy was the most resistant founder (Fig. 6). However, 53.5% of the MAGIC RILs showed high susceptibility (score > 4) to isolate 202579, compared to just 11.6% for isolate 203649 (Fig. 7). As the same phenotypic scoring scale was used to record phenotypes from infiltration and inoculation experiments using isolates 202579 and 203649, paired Wilcoxon signed-rank test was carried out. Mean scores for inoculation and infiltration using isolate 203649 were all significantly (*p* < 0.0001) lower than inoculation and infiltration results treated with isolate 202579. The distribution of inoculation phenotypic results for isolate 202579 was skewed toward susceptibility, while the results for inoculation with 203649 had most scores between 2 and 4 (Fig. 7). The phenotypic correlation between inoculation and culture filtrate infiltration experiments using the same isolate was highly significant (*p* < 0.0001) for both isolates 203649 and 202579 (Table 4). Culture filtrate infiltration with isolate 203667 was significantly correlated with glume blotch in 2016 (*r* = 0.10, *p* < 0.05) and highly significantly correlated with both infiltration (*r* = 0.70, *p* < 0.0001) and inoculation (*r* = 0.45, *p* < 0.0001) using isolate 202579 (Table 4). Isolate 203649 infiltration results were significantly correlated with leaf blotch field data in 2016 (*r* = 0.16, *p* < 0.01), 2017 (*r* = 0.09, *p* < 0.05) and 2018 (*r* = 0.11, *p* < 0.05) in Norway, while infiltration with isolate 202579 was significantly correlated with leaf blotch in Norway in 2014 (*r* = 0.18, *p* < 0.05) and 2017 (*r* = 0.13, *p* < 0.01) (Table 4). Furthermore, leaf blotch 2016 (*r* = 0.14, *p* < 0.01), 2017 (*r* = 0.13, *p* < 0.01) and 2018 (*r* = 0.13, *p* < 0.05) in Norway were significantly correlated with the seedling disease phenotypes resulting from inoculation using isolate 203649, while inoculation with isolate 202579 was significantly correlated with leaf blotch in Norway in 2014 (*r* = 0.24, *p* < 0.01), 2016 (*r* = 0.20, *p* < 0.0001) and 2017 (*r* = 0.26, *p* < 0.0001) (Table 4). Heritability (h^2^) for inoculation with isolates 203649 and 202579 was 0.31 and 0.49, respectively.

**Table 4:** Pearson correlation coefficients for corrected leaf blotch and glume blotch severities, greenhouse infiltration and inoculation

	2014NLB	2016NLB	2016NGB	2017NLB	2017NGB	2018NLB	2017ULB	2018ULB	Infil 203649	Inoc 203649	Inf 202579	Inoc 202579
Infiltration 203649	0.03	0.16**	0.00	0.09*	− 0.08	0.11*	0.06	0.06				
Inoculation 203649	0.01	0.14**	0.03	0.13**	− 0.01	0.13*	0.07	− 0.06	0.35***			
Infiltration 202579	0.18*	0.08	0.01	0.13**	0.01	0.05	0.03	0.03	0.07	0.08		
Inoculation 202579	0.24**	0.20***	0.09	0.26***	0.08	0.08	0.00	− 0.05	0.06	0.40***	0.43***	
Infiltration												
203667	0.09	0.07	0.10*	0.09	0.05	− 0.02	− 0.01	0.01	0.05	0.03	0.70***	0.45***

*N* Norway, *U* UK, *LB* leaf blotch, *GB* glume blotch. *Infil* infiltration, *Inoc* inoculation

***< 0.0001; **< 0.01; *< 0.05

### Genetic analysis of seedling experiments

Sixteen QTL on chromosomes 2A, 2D, 3A, 3B, 5B, 7B and 7D were identified via the seedling inoculation and infiltration experiments at a significance threshold of − log_10_(*p*) > 3 (Table 3; Fig. 3). Of these, eight QTL were detected for spore suspension inoculations, seven for culture filtrate infiltrations, and one for infiltration with ToxA. Among these, six QTL were significant using the more stringent significance threshold determined by permutation (listed on a trait by trait basis in Table S2), and termed here ‘strong’ QTL: (1) *QSnb.niab*-*2D.2* on chromosome 2D, detected by inoculation using both isolates 203649 and 202579 and explaining 11.42% and 4.86% of the variation, respectively. The peak markers *Excalibur_c42413_442* and *Ra_c19051_1446* at this QTL mapped to 198.36 cM and 192.18 cM on the genetic map and were located at 636 Mb and 638 Mb on the physical map (IWGSC RefSeq v1.0). (2) QTL *QSnb.niab*-*7D.1*, contributing to resistance to inoculation of isolate 203649, explained 7.89% of the variation (− log_10_(*p*) = 9.90) and was located at 69.65 cM/174 Mb on chromosome 7D. (3) *QSnb.niab*-*5B.2* on the long arm of chromosome 5B was detected via inoculation with isolate 202579 and explained 8.54% of the variation (− log_10_(*p*) = 7.93). This QTL co-located with the *Tsn1* locus identified here via infiltration with ToxA (Table 3). (4) The previously identified Tox3 effector sensitivity locus *Snn3*-*B1* on the short arm of chromosome 5B (Downie et al. 2018; Liu et al. 2009; Ruud et al. 2017) located at 6.65 Mb, co-located with QTL *QSnb.niab*-*5B.1* detected via infiltration with isolates 202579 (8.1% variation, − log_10_(*p*) = 5.48) and 203667 (10.4% variation, − log_10_(*p*) = 8.90) (Table 3).

### Haplotype analysis of *QSnb.niab*-*2A.3*

**Fig. 8:**
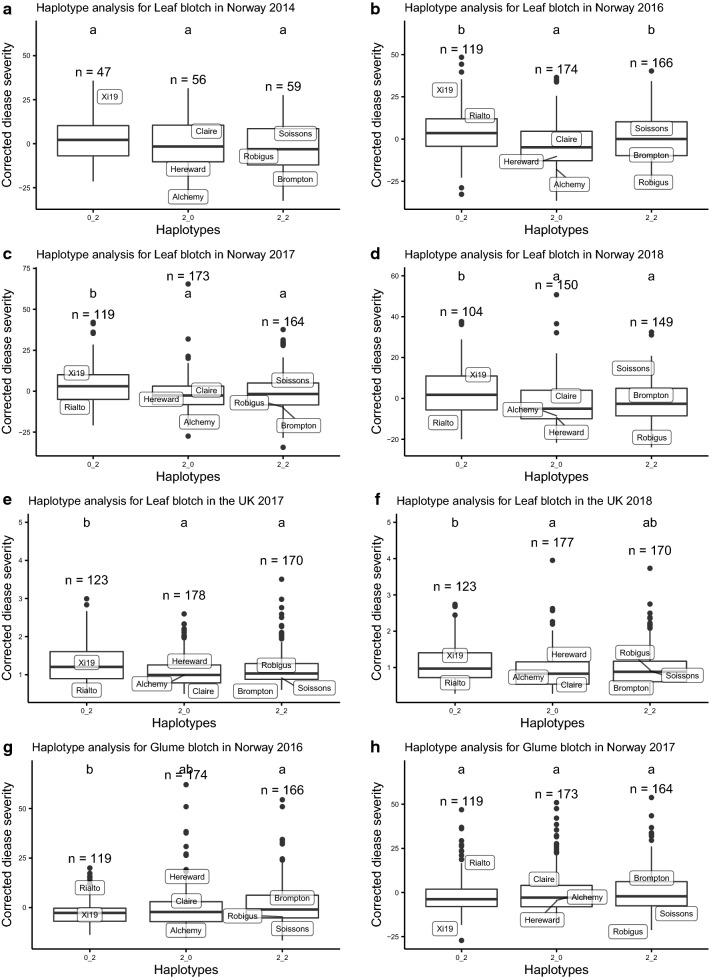
Haplotype analysis for *QSnb.niab*-*2A.3* constructed using two markers for leaf blotch in Norway (**a**–**d**), for leaf blotch in the UK (**e**, **f**) and for glume blotch in Norway (g and h), Same letter on boxplots indicates no significant difference between haplotypes determined by Kruskal–Wallis test (*p* < 0.05)

**Fig. 9:**
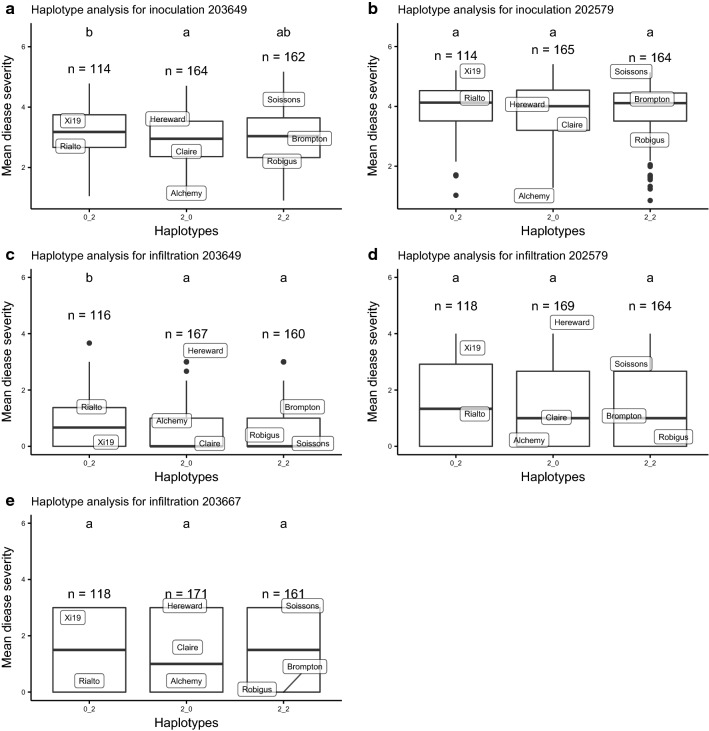
Haplotype analysis for *QSnb.niab*-*2A.3* for greenhouse inoculation experiment (**a**, **b**), greenhouse infiltration experiment (**c**–**e**). Same letter on boxplots indicates no significant difference between haplotypes determined by Kruskal–Wallis test (*p* < 0.05)

Markers *BS00062679_51* at 142.7 cM/615 Mb and *RAC875_c9372_94* at 144.8 cM/636 Mb were used to construct haplotypes at the *QSnb.niab*-*2A.3* locus, resulting in the eight founders being designated as one of three haplotypes. The corrected leaf blotch severity of haplotype 0_2 (inherited from Xi19 and Rialto) was significantly (*p* < 0.05) higher than that of haplotype 2_0 (inherited from Alchemy, Claire and Hereward). This result was consistent for all leaf blotch trials except Norway 2014, likely due to the low number of RILs tested that year (Fig. 8). The remaining haplotype 2_2 (inherited from Soissons, Brompton and Robigus) showed inconsistent resistance or susceptibility to leaf blotch in comparison with the susceptible haplotype 0_2. In contrast to the analysis of leaf blotch, haplotype 0_2 (inherited from Rialto and Xi19) was the most resistant haplotype for glume blotch in 2016 (mean corrected disease severity: − 2.44%) compared to susceptible haplotype 2_2 (mean corrected disease severity: 1.63%) although the haplotype effect was not significant in 2017 (Fig. 8h). Haplotype analysis was also carried out for phenotypic data derived from the seedling experiments (Fig. 9), with significant differences between resistant haplotype 2_0 and susceptible haplotype 0_2 observed for culture filtrate infiltration and inoculation with isolate 203649.

## Discussion

### Field inoculation methods

Naturally infected straw was used as inoculum in Norway to simulate natural infection in the field. Disease developed from the bottom to the top of the canopy. As expected, plant height and days to heading were negatively correlated with leaf blotch severity in the Norwegian trials, as reported previously (Lu and Lillemo 2014; Ruud et al. 2017). The UK field trials were infected by spraying spore suspensions derived from a single local isolate, the most common method of infection (e.g., Fried 1987; Laubscher et al. 1966; Uphaus et al. 2007; Wicki et al. 1999). The heritabilities of SNB disease severity were higher in Norway than in the UK. This might be due to various factors, including more conducive environmental conditions for pathogen infection, and the mixed local *P. nodorum* population assumed from the straw inoculation method. We used naturally infected straw as inoculum in Norway, and we would also expect variations in pathogen populations every year due to variations in climate in recent years. Therefore, relatively low but significant correlations between leaf blotch disease scores from different years could be expected given the very different agronomic environments and likely differences in *P. nodorum* isolates present between locations. Significantly, despite the contrasting inoculation methods, pathogen isolates, agronomy and geographical/environmental factors associated with these trials, we were able to identify a common QTL between sites located in Norway and the UK (*QSnb.niab*-*2A.3*). This illustrates that it is possible to identify robust field QTL for leaf blotch resistance/sensitivity that are relevant to multiple agronomic environments.

In Norway, plant height was negatively correlated to glume blotch, agreeing with previously published studies (Shatalina et al. 2014). However, in contrast to our observations for leaf blotch and with previous studies of glume blotch resistance (Aguilar et al. 2005; Wicki et al. 1999), we found days to heading to be positively correlated with glume blotch in our Norwegian trials. This might be explained by the differences between leaf blotch and glume blotch infection time, and/or different inoculation methods being used. In natural conditions, ear infection occurs later in the season compared to leaves. Thus, ears of later lines which possess relatively young tillers are usually exposed to higher infection pressure compared to early lines, as early lines mature before the disease spreads to the ears. This also explains why the mean disease severity was lower for glume blotch compared to leaf blotch: the short time in which glume blotch can develop before maturity limits the disease development. In other glume blotch studies using spray inoculation (Aguilar et al. 2005; Shatalina et al. 2014; Wicki et al. 1999), wheat ears were exposed to the pathogen directly, and the disease development was therefore less affected by the earliness of the lines. In the UK, plant height and days to heading were not found to show strong correlation with SNB. Lack of correlation with plant height in the 2018 trial may have been due to the use of plant growth regulators, following local agronomic practice. Indeed, the observation that plant height was not a significant confounding factor supports the use of local agronomic practice for the UK 2018 trial and may have helped to avoid detection of pleiotropic effects of height on the detection of SNB resistance QTL. Plant growth regulators were not deemed necessary under growth conditions in the 2017 UK trial, and no confounding effect of height was observed.

### Seedling experiments

Seedling testing was carried out to investigate whether there was any commonality between seedling and adult plant resistance. Higher mean scores for both the inoculation and infiltration results were observed for isolate 202579 compared to isolate 203649, indicating the high aggressiveness of isolate 202579. High numbers of MAGIC RILs were found to have a strong hypersensitive reaction (score 4 and 5) after inoculation using isolate 202579. This phenomenon is likely explained by more of the known NEs being produced by 202579: the isolate possess all three of the well-characterized effectors genes (*ToxA, Tox1* and *Tox3*) and the MAGIC population segregates for all three corresponding sensitivity loci (*Tsn1, Snn1* and *Snn3*-*B1*). In contrast, isolate 203649 does not produce any of these three NEs. If this isolate produces NEs, they are currently unknown, as is the allelic state of any corresponding host sensitivity loci in the MAGIC founders.

**Fig. 10:**
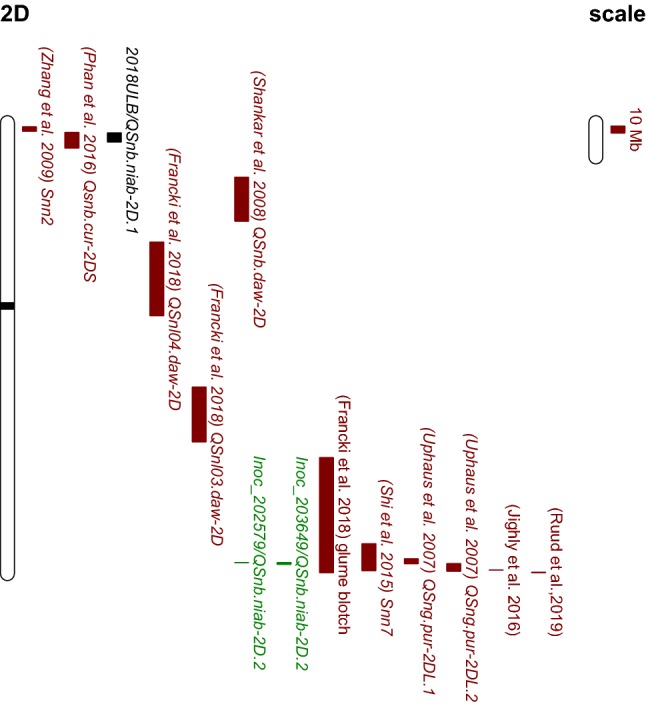
Physical map locations of QTL on chromosome 2D. QTL locations and interval sizes are indicated by bars on the right hand side of chromosome and are based on the data in Supplementary Table S4. *QSnb.niab*-*2D.1* detected for leaf blotch 2018 in the UK and *QSnb.niab*-*2D.2* detected by greenhouse inoculation in the ‘NIAB Elite MAGIC’ population. *U* UK, *LB* leaf blotch. QTL detected by this study: field leaf blotch QTL is indicated in black and seedling QTL in green. Published QTL are indicated in brown

Inoculation using both isolates 203649 and 202579 identified one QTL in common, *QSnb.niab*-*2D.2* on chromosome 2D (Fig. 10). So far, only two sensitivity loci interacting with necrotrophic effectors have been characterized on chromosome 2D. The first is the Tox2 sensitivity locus *Snn2*, located on the short arm of chromosome 2D (Zhang et al. 2009). Comparison of genetic map locations indicates that *Snn2* co-locates with the ‘weak’ leaf blotch QTL *QSnb.niab*-*2D.1* identified in the UK 2018 trial (Table S4; Fig. 10). The second is *Snn7* on the long arm of chromosome 2D, which interacts with the necrotrophic effector Tox7 (Shi et al. 2015). Various field studies have identified QTL for adult plant leaf or glume blotch resistance on wheat chromosome 2D (Aguilar et al. 2005; Francki et al. 2018; Ruud et al. 2019; Shankar et al. 2008; Uphaus et al. 2007). However, these studies mostly used relatively small mapping populations genotyped with Diversity Arrays Technology (DArT) and/or simple sequence repeat (SSR) markers, making it harder to accurately compare these QTL locations with those identified using the 90 K SNP array in this study. Nevertheless, to help facilitate QTL comparison, we anchored flanking markers for QTL from published sources to the wheat reference genome by BLASTn (Table S4). Peak markers for both isolates tested in our inoculation experiment were located within the region defined by the published *Snn7* flanking markers, between 608 to 647 Mb on chromosome 2D (Fig. 10). Interestingly, the flanking markers of previously published glume blotch resistance QTL (Francki et al. 2018; Uphaus et al. 2007) were also located within this region. Shi et al. (2015) claimed that the glume blotch resistance QTL *QSng.pur*-*2DL.1*, identified in the P92201D5 × P91193D1 population by Uphaus et al. (2007), was not *Snn7*, because none of the parent lines were sensitive to Tox7. Therefore, our QTL *QSnb.niab*-*2D.2* could be allelic to either *Snn7* or *QSng.pur*-*2DL.1.* Sensitive alleles at *Snn7* are relatively rare in wheat, found only in a few genotypes to date (Shi et al. 2015). Whether the NIAB Elite MAGIC founders carry sensitive alleles at *Snn7* is unknown, and further research would be needed to clarify this. Notably, we did not detect *QSnb.niab*-*2D.2* by culture filtrate infiltration for either of the two isolates studied here. Therefore, the underlying effector, putatively Tox7, was either not produced and/or secreted by either isolate in liquid culture, or its expression level was very low in vitro.

Another notable observation from the seedling experiments was that while infiltration with culture filtrate produced by isolate 202579 identified just the Tox3 sensitivity locus *Snn3*-*B1*, inoculation using the same isolate identified *Snn3*-*B1* and the ToxA sensitivity locus *Tsn1* (Table 3; Fig. S1 and Fig. S2). *Tsn1* was likely not detected through culture filtrate infiltration as ToxA is reported to not be expressed in vitro (Rybak et al. 2017; Tan et al. 2015). Phan et al. (2016) evaluated a population segregating for both *Snn1* and *Snn3*-*B1* using isolate SN15, which produces ToxA, Tox1 and Tox3. The wheat *Snn3*-*B1* locus was only detected in genetic analyses after knocking out the SN15 *Tox1* gene, indicating expression of Tox3 was suppressed by Tox1 in SN15. However, in our study, isolate 202579 carries all three known effector genes, *ToxA*, *Tox1* and *Tox3*. ToxA was not expressed in vitro; therefore, according to the hypothesis that *Tox3* expression is suppressed by Tox1, it might be expected that a QTL at the *Snn1* locus would be detected using culture filtrate infiltration, rather than *Snn3*-*B1* as was detected here. This may be because the expression levels of necrotrophic effectors are isolate dependent (Faris et al. 2011). The mechanism by which the effects of Tox1-*Snn1* interaction are masked by ToxA-*Tsn1* and Tox3-*Snn3*-*B1* interaction in our inoculation experiment is still unclear, but could be explained by reduced *Tox1* expression level when the pathogen interacted with the host, or epistatic effects caused by host susceptibility genes.

Isolate 203667 possessed both *ToxA* and *Tox3*. Only *Snn3*-*B1* was detected after culture filtrate infiltration, again supporting reports that ToxA is not expressed in vitro. The observation of significant correlations between culture filtrate infiltration using 203667 and infiltration/inoculation using 202579, while low correlations were found between culture filtrate infiltration using 203667 and infiltration/inoculation using 203649 (Table 4), was likely due to the similar effector profiles of isolates 203667 and 202579. Finally, *QSnb.niab*-*7D.1* is identified here as a novel QTL for SNB seedling resistance, since to our knowledge, no SNB-related QTL close to this location on chromosome 7D have previously been reported.

### QTL and haplotype analysis of field experiments

**Fig. 11:**
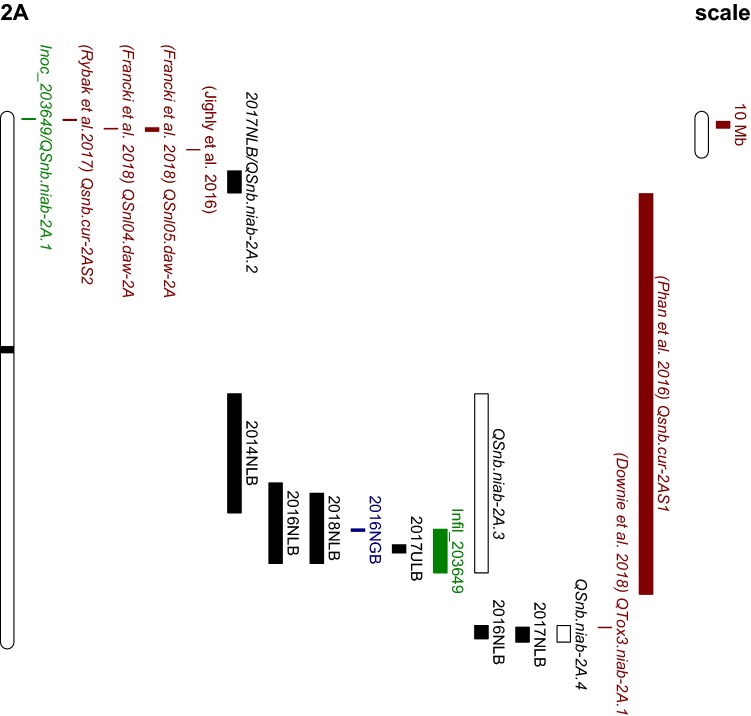
Physical map locations of QTL on chromosome 2A. QTL locations and interval sizes are indicated by bars on the right hand side of chromosome and are based on the data in Supplementary Table S3. QTL detected by this study: field leaf blotch QTL are indicated in black, field glume blotch QTL in blue (*N* Norway, *U* UK, *LB* leaf blotch, *GB* glume blotch), and seedling QTL in green (Inoc: greenhouse inoculation, Infil: greenhouse infiltration). Of these QTL, those detected in more than one environment are indicated using a white bar, along with the designated QTL name assigned in this study. Published QTL are indicated in brown

From previous studies, four QTL on chromosome 2A have been identified for SNB leaf blotch resistance/susceptibility (Francki et al. 2018; Phan et al. 2016; Rybak et al. 2017), one for glume blotch (Jighly et al. 2016) and one for Tox3 sensitivity (Downie et al. 2018). After anchoring flanking markers for these QTL to the wheat reference genome (Table S3; Fig. 11), comparison with the chromosome 2A QTL identified in this study indicated that our SNB resistance QTL *QSnb.niab*-*2A.3* (574–639 Mb) may correspond to the seedling sensitivity QTL *Qsnb.cur*-*2AS.1* identified by Phan et al. (2016). However, since the *Qsnb.cur*-*2AS.1* interval defined by SSR markers *gwm339* and *gwm312* is very large (from 112 to 709 Mb), the probability that these two QTL are the same is currently difficult to estimate. In addition, previous studies (Aguilar et al. 2005; Fried 1987; Wicki et al. 1999) showed that resistance to SNB leaf blotch and glume blotch was controlled by genetically different mechanisms. Here, we found leaf blotch and glume blotch severity in the MAGIC founders to be quite different (Fig. 1a, b), supporting the hypothesis that the genetic control of these *P. nodorum*-mediated diseases might be controlled by different genetic mechanisms. Aguilar et al. (2005) studied resistance to both leaf blotch and glume blotch in the same population by artificial inoculation, finding one QTL in common on chromosome 2B. However, this QTL was also associated with morphological traits such as heading date, flowering date and ear length. In our study, we subtracted variance caused by plant height and heading date before QTL analysis, which avoided the epistatic effects of these traits. As the QTL intervals on chromosome 2A overlapped for leaf blotch and glume blotch, we firstly considered that they represented a single QTL. However, haplotype analysis indicated that the most susceptible haplotype for leaf blotch did not show the same effect for glume blotch, suggesting the resistant mechanisms controlling those two traits might be different. Nevertheless, *QSnb.niab*-*2A.3* is a very robust QTL for leaf blotch as haplotype analysis revealed the consistency of this QTL for leaf blotch in all years and all locations, except the Norway 2014 experiment in which a much lower numbers of RILs were trialed. In addition, while *QSnb.niab*-*2A.3* was not detected in the isolate 203649 culture filtrate infiltration experiment using IM, this locus was identified as a ‘weak’ QTL via CIM, using 1, 5 and 10 cofactors (Table 3). Furthermore, haplotype analysis showed significant increase in disease severity associated with the haplotype found to increase field SNB susceptibility (Fig. 9a, c). Therefore, isolate 203649 may produce an unknown effector which interacts with the susceptible allele underlying the *QSnb.niab*-*2A.3* haplotype. Significant correlations were observed between the infiltration experiment for isolate 203649 and leaf blotch in Norway for three years (2016–2018), while infiltration with isolate 202579 was significantly correlated with two years (2014 and 2017) and infiltration using 203667 was not significantly correlated with any leaf blotch field data. This indicates that even though isolate 203649 is less aggressive than 202579 under greenhouse conditions, the unknown effector(s) produced by this isolate may still play an important role in the field. Nevertheless, low correlations on average were found between seedling inoculation/infiltration and leaf blotch field data, indicating genetic control of SNB resistance is largely controlled by different genes/pathways between these growth stages in MAGIC lines. However, the identification of *QSnb.niab*-*2A.3* via seedling and field testing indicates that at least some genetic components are in common. One possible reason for an overall lack of strong correlation is that the *P. nodorum* isolates used for seedling screens might not be the most representative isolates of the local *P. nodorum* population in the field. Another possible reason would be that some of the field resistances/susceptibility could not be fully explained by NE-*Snn* interactions, as up to now, only four such interactions have been found contributing to field resistances/susceptibility (Friesen et al. 2009; Phan et al. 2016; Ruud and Lillemo 2018). Other underexplored plant resistant mechanisms may be involved in field SNB resistance.

Anchoring *QSnb.niab*-*2A.4* peak markers to the 2A physical map (758–759 Mb) found it to overlap with the previously identified minor Tox3 sensitivity QTL, *QTox3.niab*-*2A.1* (Table S3) (Downie et al. 2018). However, we did not detect the major Tox3 sensitivity locus *Snn3*-*B1* in the field, while *QSnb.niab*-*2A.4* was one of the major fields QTL identified, detected in two out of the four years investigated. *QSnb.niab*-*2A.4* was also detected as a ‘weak’ QTL via inoculation using isolate 203649 (Table 3). The QTL *QSnb.niab*-*3A*, anchored using peak marker *wsnp_Ex_c6833_11782875* to 10 Mb on chromosome 3A (Table 3; Fig. S4), represents a previously unreported QTL for SNB leaf blotch resistance. Here, the QTL was detected in two field seasons in Norway (− log_10_(*p*) > 3), as well as via culture filtrate infiltration using isolate 202579 (− log_10_(*p*) > 3) indicating *P. nodorum* effector(s) may play a role in controlling field SNB sensitivity for this QTL. Additional QTL on chromosomes 2A, 2D and 6A were identified as potentially co-locating with previously reported QTL (Table 3); however, they were only ‘weakly’ significant and identified in just one environment in our study (Table 3). For example, the ‘weak’ QTL *QSnb.niab*-*6A.2* identified for glume blotch in 2016 collocated with the previously reported sensitivity locus *Snn6* (Table S5) (Gao et al. 2015; Arseniuk et al. 2004). Finally, *QSnb.niab*-*6A.1* has not previously been reported and therefore represents a novel QTL for leaf blotch disease resistance under field conditions (Table 3 and S6).

The finding that *QSnb.niab*-*2A.3* haplotype 0_2 for higher leaf blotch severity was associated with increased sensitivity to culture filtrate from strain 203649 compared to haplotype 2_0, indicates this QTL for field SNB resistance/susceptibility may be controlled by a previously undescribed NE-*Snn* interaction, making this a target for identification of the underlying gene(s) in both the pathogen and host. Similarly, identification of *QSnb.niab*-*3A* in the field as well as via culture filtrate infiltration indicates that this too may represent a new NE-*Snn* interaction. Development of diagnostic Kompetitive Allele-Specific PCR (KASP) markers (Semagn et al. 2014) for field SNB resistance/culture filtrate insensitivity could allow marker assisted selection for beneficial alleles in order to breed new wheat varieties with increased resistance to SNB leaf blotch. Furthermore, the observation that these field-relevant QTL are also detected via seedling culture filtrate infiltration indicates it could be possible to further refine the downstream genetic analyses of this QTL via seedling screens of progeny derived from crosses between near isogenic line pairs developed for each QTL, greatly simplifying the logistics of screening for genetic recombinants within the QTL interval. Combining such seedling phenotyping with approaches such as ‘speed breeding’ (Watson et al. 2018) may greatly reduce experimental timelines for future map-based cloning of the gene(s) underlying *QSnb.niab*-*2A.3* and *QSnb.niab*-*3A*.

## Electronic supplementary material

Below is the link to the electronic supplementary material.

Supplementary material 1 (DOCX 55 kb)

Fig. S1 Genetic map of the *Snn3*-*B1* locus detected by greenhouse experiment on the short arm of chromosome 5B (*QSnb.niab*-*5B.1)* in the NIAB Elite MAGIC population. Peak markers are indicated in pink. (PDF 75 kb)

Fig. S2 Genetic map of the *Tsn1* locus detected by seedling inoculation using isolate 202579 on the long arm of chromosome 5B (*QSnb.niab*-*5B.2*) in the ‘NIAB Elite MAGIC’ population. Peak markers are indicated in pink. (PDF 78 kb)

Fig. S3 Genetic map of the *QSnb.niab*-*7D.1* and *QSnb.niab*-*7D.2* loci, detected by infiltration of isolate 203649, and *QSnb.niab*-*7D.3* detected by leaf blotch 2016 in Norway on chromosome 7D in the ‘NIAB Elite MAGIC’ population. N: Norway, LB: leaf blotch, Peak markers are indicated in pink. (PDF 92 kb)

Fig. S4 Genetic map of the *QSnb.niab*-*3A* locus on the short arm of chromosome 3A in the ‘NIAB Elite MAGIC’ population. N: Norway, LB: leaf blotch. Peak markers are indicated in pink. (PDF 106 kb)

Fig. S5 Genetic map of the *QSnb.niab*-*6A.1* and *QSnb.niab*-*6A.2* loci, detected by glume blotch 2016 in Norway on chromosome 6A in the ‘NIAB Elite MAGIC’ population. N: Norway, LB: leaf blotch, GB: glume blotch. Peak markers and *Snn6* markers (Gao et al. 2015) are indicated in pink. (PDF 87 kb)

Fig. S6 Corrected disease severity of MAGIC founders in different years and locations. (a) Leaf blotch severity in Ås, Norway, (b) glume blotch severity in Ås, Norway. (PDF 5 kb)

Supplementary material 8 (XLSX 121 kb)

## Data Availability

(1) The population used in current study was described by Mackay et al. (2014) and Gardner et al. (2016), with the raw genotype data also available via http://www.niab.com/pages/id/326/Resources/. (2) Phenotypic data generated during this study are included in this published article [and its supplementary information files].
