# Supplementary material for: Genetic mapping using a wheat multi-founder population reveals a locus on chromosome 2A controlling resistance to both leaf and glume blotch caused by the necrotrophic fungal pathogen Parastagonospora nodorum
Source: Theor Appl Genet. 2020 Jan 29;133(3):785–808. doi: 10.1007/s00122-019-03507-w (PMC7021668; doi:10.1007/s00122-019-03507-w)
Supplement: Supplementary file 1 — Supplementary material 1 (DOCX 55 kb) [file 122_2019_3507_MOESM1_ESM.docx]

**Table S1** Heritability (h^2^) of leaf blotch severity N: Norway, U: UK, LB: leaf blotch, GB: glume blotch.

| Environment | Heritability (%) |
| --- | --- |
| 2014NLB | 73.59 |
| 2016NLB | 77.45 |
| 2017NLB | 48.00 |
| 2018NLB | 57.49 |
| 2017ULB | 13.61 |
| 2018ULB | 57.25 |
| 2016NGB | 59.56 |
| 2017NGB | 45.64 |

**Table S2** QTL significance thresholds calculated by permutation for each environment.

| Environment | Location | Threshold (-log10P) |
| --- | --- | --- |
| 2014 Leaf blotch | Field, Norway | 4.64 |
| 2016 Leaf blotch | Field, Norway | 4.39 |
| 2016 Glume blotch | Field, Norway | 4.09 |
| 2017 Leaf blotch | Field, Norway | 4.28 |
| 2017 Glume blotch | Field, Norway | 4.42 |
| 2018 Leaf blotch | Field, Norway | 4.63 |
| 2017 Leaf blotch | Field, UK | 4.41 |
| 2018 Leaf blotch | Field, UK | 4.23 |
| Infiltration 202579 | Greenhouse | 4.22 |
| Inoculation 202579 | Greenhouse | 4.43 |
| Infiltration 203649 | Greenhouse | 4.34 |
| Inoculation 203649 | Greenhouse | 4.41 |
| Infiltration 203667 | Greenhouse | 4.49 |

**Table S3** Overview of QTL identified by published SNB studies on wheat chromosome 2A., based on the wheat reference genome (RefSeq v1.0). The genomic locations of the peak SNPs have been included and shown in bold. Marker used for constructing haplotype analysis are shown bold and underlined. N: Norway, LB: leaf blotch, left: left flanking marker of the QTL, right: right flanking marker of QTL, peak: peak marker of QTL.

| Marker ID | Population | Physical position start (bp) | Physical position end (bp) | Reference | Source/gene function |
| --- | --- | --- | --- | --- | --- |
| RAC875_c44680_90 | NIAB Elite MAGIC | 820543 | 820443 | This study/ *QSnb.niab-2A.1* | 203649_inoculation_left |
| wmc382a | Calingiri × Wyalkatchem | 2320465 | 2320686 | (Rybak et al. 2017)/ *Qsnb.cur-2AS2* | Seedling leaf blotch |
| **BS00111318_51** | NIAB Elite MAGIC | 2378473 | 2378574 | This study*/ QSnb.niab-2A.1* | 203649_inoculation_peak |
| barc124a | Calingiri × Wyalkatchem | 3784350 | 3784367 | (Rybak et al. 2017)/ *Qsnb.cur-2AS2* | Seedling leaf blotch |
| IWB29103 | P92201D5 × P91193D1 | 14815405 | 14815505 | (Francki et al. 2018)/ *Qsnl05.daw-2A* | Adult plant leaf blotch |
| IWB32474 | P92201D5 × P91193D1 | 15608826 | 15608891 | (Francki et al. 2018)/ *Qsnl04.daw-2A* | Adult plant leaf blotch |
| IWB9206 | P92201D5 × P91193D1 | 16321845 | 16321745 | (Francki et al. 2018)/ *Qsnl04.daw-2A* | Adult plant leaf blotch |
| IWB22268 | P92201D5 × P91193D1 | 19649717 | 19649817 | (Francki et al. 2018)/ *Qsnl05.daw-2A* | Adult plant leaf blotch |
| wPt-1657 | GWAS of 320 synthetic hexaloid wheat (SHW) lines | 47152165 | 47152854 | (Jighly et al. 2016) | Glume blotch |
| Kukri_c24852_466 | NIAB Elite MAGIC | 78844360 | 78844461 | This study/ *QSnb.niab-2A.2* | 2017NLB-left |
| **Excalibur_c637_1078** | NIAB Elite MAGIC | 90029248 | 90029349 | This study/ *QSnb.niab-2A.2* | 2017NLB-peak |
| BS00008805_51 | NIAB Elite MAGIC | 111457270 | 111457371 | This study/ *QSnb.niab-2A.2* | 2017NLB-right |
| gwm339 | Calingiri × Wyalkatchem | 112746128 | 112746147 | (Phan et al. 2016)/ *Qsnb.cur.2AS1* | Seedling leaf blotch |
| JD_c2056_506 | NIAB Elite MAGIC | 410872779 | 410872879 | This study/ *QSnb.niab-2A.3* | 2014NLB-left |
| **BS00055514_51** | NIAB Elite MAGIC | 543625443 | 543625544 | This study/ *QSnb.niab-2A.3* | 2014NLB-peak/2016NLB-left |
| Excalibur_c1793_97 | NIAB Elite MAGIC | 558953000 | 558953100 | This study/ *QSnb.niab-2A.3* | 2018NLB-left |
| Kukri_c7825_288 | NIAB Elite MAGIC | 588258005 | 588258104 | This study/ *QSnb.niab-2A.3* | 2014NLB-right |
| **Ku_c5710_312** | NIAB Elite MAGIC | 605800158 | 605800259 | This study/ *QSnb.niab-2A.3* | 2016NLB-peak |
| RAC875_c20247_398 | NIAB Elite MAGIC | 611946675 | 611946776 | This study/ *QSnb.niab-2A.3* | 2016NGB-left |
| BS00022641_51 |  | 612422267 | 612422167 |  | 203649_infiltration_left |
| **BS00062679_51** | NIAB Elite MAGIC | 615287656 | 615287757 | This study/ *QSnb.niab-2A.3* | 2016NGB-peak/right |
| **RAC875_c9372_94** | NIAB Elite MAGIC | 635606921 | 635606992 | This study/ *QSnb.niab-2A.3* | 2018NLB-peak/2017ULB-peak&left |
| BS00012320_51 | NIAB Elite MAGIC | 647927316 | 647927416 | This study/ *QSnb.niab-2A.3* | 2017ULB-right |
| **BS00090569_51** | NIAB Elite MAGIC | 653680962 | 653680862 | This study/ *QSnb.niab-2A.3* | 203649_infiltration_ peak |
| BS00022241_51 | NIAB Elite MAGIC | 663328916 | 663329017 | This study/ *QSnb.niab-2A.3* | 2016NLB-right /2018NLB-right |
| IAAV4015 | NIAB Elite MAGIC | 677529636 | 677529836 | This study/ *QSnb.niab-2A.3* | 203649_infiltration_ right |
| gwm312 | Calingiri × Wyalkatchem | 709048504 | 709048682 | (Phan et al. 2016)/ *Qsnb.cur.2AS1* | Seedling leaf blotch |
| wsnp_Ra_c6586_11477949 | NIAB Elite MAGIC | 755929525 | 755929615 | This study/ *QSnb.niab-2A.4* | 2016NLB-left |
| **Excalibur_c4372_363** | NIAB Elite MAGIC | 758396871 | 758396972 | This study/ *QSnb.niab-2A.4* | 2017NLB-peak |
| JD_c11825_1135 | NIAB Elite MAGIC | 758397388 | 758397464 | This study/ *QSnb.niab-2A.4* | 2017NLB -left |
| Excalibur_c20478_641 | NIAB Elite MAGIC | 758554464 | 758554565 | (Downie et al. 2018)/ *QTox3.niab2A.1* | Tox3-infiltration |
| BS00070979_51 | NIAB Elite MAGIC | 758692088 | 758692189 | (Downie et al. 2018)/ *QTox3.niab2A.1* | Tox3-infiltration |
| **wsnp_Ra_c17622_26522072** | NIAB Elite MAGIC | 759489122 | 759489310 | This study/ *QSnb.niab-2A.4* | 2016NLB -peak |
| BS00022252_51 | NIAB Elite MAGIC | 775619839 | 775619939 | This study/ *QSnb.niab-2A.4* | 2016NLB-right |
| Tdurum_contig8350_350 | NIAB Elite MAGIC | 780714571 | 780714672 | This study/ *QSnb.niab-2A.4* | 2017NLB -right |

**Table S4** Overview of QTL identified by published SNB studies on wheat chromosome 2D, based on the wheat reference genome (RefSeq v1.0). The genomic locations of the peak SNPs have been included and shown in bold. N: Norway, U: UK, LB: leaf blotch, GB: glume blotch. left: left flanking marker of the QTL, right: right flanking marker of QTL, peak: peak marker of QTL.

| Marker ID | Population | Physical position start (bp) | Physical position end (bp) | Reference | Source |
| --- | --- | --- | --- | --- | --- |
| Xcfd56 | BR34×Grandin | 6158983 | 6158963 | (Zhang et al. 2009) | *Snn2* |
| Xcfd51 | BR34×Grandin | 12360665 | 12360684 | (Zhang et al. 2009) | *Snn2* |
| cfd36 | Calingiri × Wyalkatchem | 14362782 | 14362981 | (Phan et al. 2016)/ *Qsnb.cur-2DS* |  |
| **BS00029208_51** | NIAB Elite MAGIC | 14897896 | 14897996 | This study/ *QSnb.niab-2D.1* | 2017ULB-peak |
| wsnp_JD_rep_c63957_40798083 | NIAB Elite MAGIC | 20768482 | 20768682 | This study/ *QSnb.niab-2D.1* | 2017ULB -left |
| BobWhite_c59161_181 | NIAB Elite MAGIC | 27859904 | 27859806 | This study/ *QSnb.niab-2D.1* | 2017ULB -right |
| wPt-669517 | Calingiri × Wyalkatchem | 37053347 | 37053740 | (Phan et al. 2016)/ *Qsnb.cur-2DS* |  |
| cfd11 | WAWHT2074 × 6HRWSN125 | 79231376 | 79231506 | (Shankar et al. 2008)/ *QSnb.daw-2D* | Adult plant leaf blotch |
| gwm30 | WAWHT2074 × 6HRWSN125 | 142336641 | 142336663 | (Shankar et al. 2008)/ *QSnb.daw-2D* | Adult plant leaf blotch |
| IWA8151 | WAWHT2074 × 6HRWSN125 | 173007798 | 173007990 | (Francki et al. 2018)/ *Qsnl04.daw-2D* | Adult plant leaf blotch |
| IWB38724 | WAWHT2074 × 6HRWSN125 | 279049474 | 279049410 | (Francki et al. 2018)/ *Qsnl04.daw-2D* | Adult plant leaf blotch |
| Excalibur_c4248_1411 | NIAB Elite MAGIC | 316144729 | 316144828 | This study/ *QSnb.niab-2D.2* | 202579_inoculation-left |
| IWB46396 | WAWHT2074 × 6HRWSN125 | 382170351 | 382170451 | (Francki et al. 2018)/ *Qsnl03.daw-2D* | Adult plant leaf blotch |
| IWB38687 | WAWHT2074 × 6HRWSN125 | 461301212 | 461301312 | (Francki et al. 2018)/ *Qsnl03.daw-2D* | Adult plant leaf blotch |
| IWB31450 | P92201D5 × P91193D1 | 484328365 | 484328343 | (Francki et al. 2018) | Glume blotch |
| cfd44 | Chinese Spring × Timstein | 608561046 | 608633134 | (Shi et al. 2015) | *Snn7* |
| Xgwm526.1 | P92201D5 × P91193D1 | 630399094 | 630399233 | (Uphaus et al. 2007)/ *Qng.pur-2DL.1* | Glume blotch |
| **Excalibur_c42413_442** | NIAB Elite MAGIC | 635950166 | 635950267 | This study/ *QSnb.niab-2D.2* | 203649_inoculation-  peak |
| BS00010685_51 | NIAB Elite MAGIC | 635950569 | 635950669 | This study/ *QSnb.niab-2D.2* | 202579_inoculation-right |
| Kukri_c36328_419 | NIAB Elite MAGIC | 635951859 | 635951940 | This study/ *QSnb.niab-2D.2* | 203649_inoculation-right |
| Xcfd50 | P92201D5 × P91193D1 | 637443897 | 637444139 | (Uphaus et al. 2007)/ *QSng.pur-2DL.1/ QSng.pur-2DL.2* | Glume blotch |
| RFL_Contig1128_620 | NIAB Elite MAGIC | 638147672 | 638147754 | This study/ *QSnb.niab-2D.2* | 203649_inoculation-left |
| **Ra_c19051_1446** | NIAB Elite MAGIC | 638147672 | 638147754 | This study/ *QSnb.niab-2D.2* | 202579_inoculation-  peak |
| wPt-7825 | GWAS of 320 synthetic hexaloid wheat (SHW) lines | 646772948 | 646773564 | (Jighly et al. 2016) | Glume blotch |
| gwm311 | Chinese Spring × Timstein | 647509839 | 647509858 | (Shi et al. 2015) | *Snn7* |
| wPt-9848 | P92201D5 × P91193D1 | 648059097 | 648058258 | (Uphaus et al. 2007)/*QSng.pur-2DL.2* | Glume blotch |
| Excalibur_c31806_912 | GWAS of 121 spring lines | 649815122 | 649815222 | (Ruud et al, 2019 in press) | Adult plant leaf blotch |
| BS00015680_51 | GWAS of 121 spring lines | 650322729 | 650322629 | (Ruud et al, 2019 in press) | Adult plant leaf blotch |
| IAAV1322 | GWAS of 121 spring lines | 650322873 | 650322991 | (Ruud et al, 2019 in press) | Adult plant leaf blotch |
| Excalibur_rep_c67599_2154 | GWAS of 121 spring lines | 650325174 | 650325274 | (Ruud et al, 2019 in press) | Adult plant leaf blotch |
| IAAV6032 | GWAS of 121 spring lines | 650325193 | 650325352 | (Ruud et al, 2019 in press) | Adult plant leaf blotch |
| IWB21124 | P92201D5 × P91193D1 | 650326781 | 650326881 | (Francki et al. 2018) | Glume blotch |
| Excalibur_rep_c67599_242 | GWAS of 121 spring lines | 650327186 | 650327086 | (Ruud et al, 2019 in press) | Adult plant leaf blotch |

**Table S5** Overview of QTL identified by published SNB studies on wheat chromosome 6A, based on the wheat reference genome (RefSeq v1.0). The genomic locations of the peak SNPs have been included and shown in bold. N: Norway, U: UK, LB: leaf blotch, GB: glume blotch. Left: left flanking marker of the QTL, right: right flanking marker of QTL, peak: peak marker of QTL.

| Marker ID | Population | Physical position start (bp) | Physical position end (bp) | Reference | Source |
| --- | --- | --- | --- | --- | --- |
| IAAV5188 | NIAB Elite MAGIC | 74025753 | 74025954 | This study/ *QSnb.niab-6A.1* | 2016NLB- Left |
| **TA004558_1018** | NIAB Elite MAGIC | 97809626 | 97809680 | This study/ *QSnb.niab-6A.1* | 2016NLB -peak |
| RFL_Contig3088_949 | NIAB Elite MAGIC | 249160604 | 249160705 | This study/ *QSnb.niab-6A.1* | 2016NLB- right |
| BE403326 | W-7984 × Opata 85 | 574221943 | 574221462 | (Gao et al. 2015) | *Snn6* |
| gwm570 | Alba × Begra | 579125838 | 579125980 | (Arseniuk et al. 2004) | Seedling leaf |
| mwg934 | Alba × Begra | 583269284 | 583269303 | (Arseniuk et al. 2004) | Seedling leaf |
| **GENE_4028_152** | NIAB Elite MAGIC | 600395629 | 600395722 | This study/ *QSnb.niab-6A.2* | 2016NGB-Peak |
| BS00096240_51 | NIAB Elite MAGIC | 600406107 | 600406208 | This study/ *QSnb.niab-6A.2* | 2016NGB - right |
| BF428729 | W-7984 × Opata 85 | 603135924 | 603136145 | (Gao et al. 2015) | *Snn6* |
| BE424987 | W-7984 × Opata 85 | 606979803 | 606979976 | (Gao et al. 2015) | *Snn6* |

**Table S6** Plant height (PH) QTL identified in the ‘NIAB Elite MAGIC’ population from field trials conducted in Norway in 2014, 2016, 2017 and 2018, and the UK in 2017 and 2018. QTL with –log_10_(p) value > 3 are presented, with QTL above the permutated trait-specific significance threshold highlighted in bold. Chromosome (Chr.), proportion of the variance explained by QTL (R^2^). The –log10(p) values coded as ‘Inf’ are due to a p-value of 0, resulting in an error when converted to the log_10_ scale. ^†^ Genetic map: Gardner et al. (2016).

| QTL | Location | Trait | Year | Chr | Interval (cM)^†^ | Flanking markers | Peak Marker | -log_10_ (p) | R^2^ (%) | Detected by QTL methods |
| --- | --- | --- | --- | --- | --- | --- | --- | --- | --- | --- |
| ***QPh.niab-2D*** | **UK** | **PH** | **2017** | **2D** | **52.86-62.71** | **BS00011425_51 and wsnp_CAP12_c1503_764765** | **wsnp_CAP12_c1503_764765** | **7.96** | **7.19** | **IM, CIM (cov5, cov10),IBD** |
| ***QPh.niab-4B*** | **Norway** | **PH** | **2014** | **4B** | **38.66-57.22** | **wsnp_CAP11_c1103_647926 and BS00076259_51** | **BS00023766_51** | **13.74** | **18.36** | **IM, CIM (cov5, cov10),IBD** |
| ***QPh.niab-4B*** | **Norway** | **PH** | **2016** | **4B** | **40.17-57.22** | **BS00022534_51 and BS00076259_51** | **RAC875_c27536_611** | **Inf** | **6.99** | **IM, CIM (cov5, cov10),IBD** |
| ***QPh.niab-4B*** | **Norway** | **PH** | **2017** | **4B** | **47.14-57.22** | **BS00100838_51 and BS00076259_51** | **RAC875_c27536_611** | **Inf** | **7.24** | **IM, CIM (cov5, cov10),IBD** |
| ***QPh.niab-4B*** | **Norway** | **PH** | **2018** | **4B** | **47.14-57.22** | **BS00100838_51 and BS00076259_51** | **BS00021984_51** | **11.50** | **5.54** | **IM, CIM (cov5, cov10),IBD** |
| ***QPh.niab-4B*** | **UK** | **PH** | **2017** | **4B** | **38.66-57.22** | **wsnp_CAP11_c1103_647926 and BS00076259_51** | **RAC875_c27536_611** | **Inf** | **7.18** | **IM, CIM (cov5, cov10),IBD** |
| ***QPh.niab-4B*** | **UK** | **PH** | **2018** | **4B** | **47.14-57.22** | **BS00100838_51 and BS00076259_51** | **RAC875_c27536_611** | **11.73** | **5.86** | **IM, CIM (cov5, cov10),IBD** |
| ***QPh.niab-4D*** | **Norway** | **PH** | **2014** | **4D** | **24.93-42.63** | **Kukri_rep_c68594_530 and Excalibur_c53541_723** | **RHT2** | **Inf** | **32.38** | **IM, CIM (cov5, cov10),IBD** |
| ***QPh.niab-4D*** | **Norway** | **PH** | **2016** | **4D** | **24.93-42.13** | **Kukri_rep_c68594_530 and Excalibur_c23163_98** | **RAC875_c1673_663** | **Inf** | **17.55** | **IM, CIM (cov5, cov10),IBD** |
| ***QPh.niab-4D*** | **Norway** | **PH** | **2017** | **4D** | **24.93-42.13** | **Kukri_rep_c68594_530 and Excalibur_c23163_98** | **RAC875_c1673_663** | **Inf** | **17.82** | **IM, CIM (cov5, cov10),IBD** |
| ***QPh.niab-4D*** | **Norway** | **PH** | **2018** | **4D** | **24.93-42.63** | **Kukri_rep_c68594_530 and Excalibur_c53541_723** | **RHT2** | **Inf** | **13.81** | **IM, CIM (cov5, cov10),IBD** |
| ***QPh.niab-4D*** | **UK** | **PH** | **2017** | **4D** | **29.93-42.13** | **Kukri_rep_c68594_530 and Excalibur_c23163_98** | **RAC875_c1673_663** | **Inf** | **19.48** | **IM, CIM (cov5, cov10),IBD** |
| ***QPh.niab-4D*** | **UK** | **PH** | **2018** | **4D** | **24.93-42.13** | **Kukri_rep_c68594_530 and Excalibur_c23163_98** | **RAC875_c1673_663** | **Inf** | **14.77** | **IM, CIM (cov5, cov10),IBD** |
| *QPh.niab-6A.1* | Norway | PH | 2016 | 6A | 0-8.63 | BobWhite_c15849_189 and RAC875_c6135_95 | Excalibur_c18072_214 | 3.30 | 4.82 | IM, CIM (cov5, cov10),IBD |
| *QPh.niab-6A.2* | UK | PH | 2017 | 6A | 88.28-103.35 | wsnp_Ex_c31149_39976103 and Tdurum_contig50698_601 | Kukri_c27958_334 | 3.09 | 3.4 | IM, CIM (cov5, cov10) |

**Table S7** Days to heading (DH) QTL identified in the ‘NIAB Elite MAGIC’ population from field trials conducted in Norway in 2014, 2016, 2017 and 2018, and the UK in 2017 and 2018. QTL with –log_10_(p) value > 3 are presented, with QTL above the permutated trait-specific significance threshold highlighted in bold. Chromosome (Chr.), proportion of the variance explained by QTL (R^2^). The –log10(p) values coded as ‘Inf’ are due to a p-value of 0, resulting in an error when converted to the log_10_ scale. ^†^ Genetic map: Gardner et al. (2016).

| QTL | Location | Trait | Year | Chr | Interval (cM)^†^ | Flanking markers | Peak Marker | -log_10_ (p) | R^2^ (%) | Detected by QTL methods |
| --- | --- | --- | --- | --- | --- | --- | --- | --- | --- | --- |
| ***QDh.niab-1B*** | **Norway** | **DH** | **2017** | **1B** | **341.89-351.10** | **RAC875_c28629_101 and Kukri_c44587_130** | **BS00027006_51** | **7.55** | **6.43** | **IM, CIM (cov5, cov10), IBD** |
| ***QDh.niab-2A*** | **Norway** | **DH** | **2014** | **2A** | **140.76-159.02** | **Kukri_c24064_2095 and BS00065865_51** | **IAAV4015** | **4.55** | **13.15** | **IM, CIM (cov5, cov10)** |
| *QDh.niab-2B* | UK | DH | 2018 | 2B | 160.78-172.37 | wsnp_Ex_c3044_5620102 and Ra_c23048_474 | Tdurum_contig19413_656 | 3.50 | 4.13 | **IM, CIM (cov5, cov10), IBD** |
| ***QDh.niab-2D*** | **Norway** | **DH** | **2016** | **2D** | **50.84-62.71** | **BobWhite_c59161_181 and wsnp_CAP12_c1503_764765** | **RAC875_c7319_195** | **5.02** | **6.11** | **IM, CIM (cov5, cov10), IBD** |
| ***QDh.niab-2D*** | **Norway** | **DH** | **2017** | **2D** | **50.84-62.71** | **BobWhite_c59161_181 and wsnp_CAP12_c1503_764765** | **RAC875_c7319_195** | **6.73** | **5.71** | **IM, CIM (cov5, cov10), IBD** |
| ***QDh.niab-2D*** | **UK** | **DH** | **2017** | **2D** | **50.84-62.71** | **BobWhite_c59161_181 and wsnp_CAP12_c1503_764765** | **BS00064538_51** | **Inf** | **37.55** | **IM, CIM (cov5, cov10), IBD** |
| ***QDh.niab-2D*** | **UK** | **DH** | **2018** | **2D** | **60.67**  **-62.71** | **BS00011425_51 and wsnp_CAP12_c1503_764765** | **wsnp_CAP12_c1503_764765** | **6.31** | **6.86** | **IM, CIM (cov5, cov10), IBD** |
| ***QDh.niab-3A*** | **Norway** | **DH** | **2016** | **3A** | **197.83-214.37** | **Ex_c15087_564 and BS00039498_51** | **GENE_1549_110** | **4.33** | **3.56** | **IM, CIM (cov10)** |
| ***QDh.niab-4D*** | **Norway** | **DH** | **2016** | **4D** | **0-26.97** | **BS00054978_51 and RAC875_c6922_291** | **Excalibur_c26088_184** | **5.06** | **4.98** | **IM, CIM (cov5, cov10), IBD** |
| ***QDh.niab-5A*** | **Norway** | **DH** | **2016** | **5A** | **167.30-183.69** | **IAAV5294 and BS00067209_51** | **wsnp_Ra_c17216_26044790** | **5.73** | **4.99** | **IM, CIM (cov5, cov10), IBD** |
| *QDh.niab-5D* | Norway | DH | 2018 | 5D | 124.29-133.32 | BS00033770_51 and BS00067651_51 | BS00067651_51 | 3.42 | 5.02 | IM, CIM (cov5, cov10), IBD |
| *QDh.niab-6A* | Norway | DH | 2014 | 6A | 124.90-142.54 | wsnp_BG262421A_Ta_2_2 and Tdurum_contig78006_158 | RAC875_c23305_563 | 3.52 | 10.5 | IM, CIM (cov5, cov10) |
| *QDh.niab-6B* | Norway | DH | 2016 | 6B | 206.35-217.64 | IACX2322 and BS00068245_51 | Excalibur_c57840_227 | 3.64 | 3.5 | IM, CIM (cov5) |

**Table S8** Uncorrected leaf blotch severity (uLB) QTL identified in the ‘NIAB Elite MAGIC’ population from field trials conducted in Norway in 2014, 2016, 2017 and 2018, and the UK in 2017 and 2018. QTL with –log_10_(p) value > 3 are presented, with QTL above the permutated trait-specific significance threshold highlighted in bold. Chromosome (Chr.), proportion of the variance explained by QTL (R2). ^†^ Genetic map: Gardner et al. (2016).

| QTL | Location | Trait | Year | Chr | Interval (cM) ^†^ | Flanking markers | Peak Marker | -log_10_ (p) | R^2^ (%) | Detected by QTL methods |
| --- | --- | --- | --- | --- | --- | --- | --- | --- | --- | --- |
| ***QSnb.niab-2A.3*** | **Norway** | **uLB** | **2014** | **2A** | **121.07-140.25** | **JD_c2056_506 and BS00022903_51** | **BS00059475_51** | **5.49** | **16.22** | **IM, CIM (cov5, cov10), IBD** |
| ***QSnb.niab-2A.3*** | **Norway** | **uLB** | **2016** | **2A** | **129.14-146.83** | **BobWhite_c1049_338 and BS00022241_51** | **Ku_c5710_312** | **5.30** | **6.63** | **IM, CIM (cov5, cov10), IBD** |
| *QSnb.niab-2A.3* | Norway | uLB | 2018 | 2A | 127.63-146.83 | RAC875_c15213_1942 and BS00022241_51 | RAC875_c9372_94 | 4.45 | 6.11 | IM, CIM (cov5, cov10), IBD |
| *QSnb.niab-2A.4* | Norway | uLB | 2017 | 2A | 227.51-241.18 | BS00022321_51 and Kukri_c50116_644 | Excalibur_c4372_363 | 3.76 | 4.93 | IM, CIM (cov5, cov10) |
| ***QSnb.niab-3A*** | **Norway** | **uLB** | **2017** | **3A** | **2.01-34.65** | **Tdurum_contig50376_375 and RAC875_c371_251** | **BS00023189_51** | **4.35** | **4.52** | **IM, CIM (cov5, cov10)** |
| *QSnb.niab-4A.2* | Norway | uLB | 2016 | 4A | 129.70-141.35 | BS00099725_51 and RAC875_c35819_165 | BS00012482_51 | 3.38 | 4.19 | IM, CIM (cov5, cov10), IBD |
| *QSnb.niab-5B.3* | Norway | uLB | 2014 | 5B | 76.62-94.35 | RAC875_c24376_704 and wsnp_Ex_rep_c68003_66744451 | BS00066138_51 | 3.06 | 10.16 | IM, CIM (cov5, cov10) |
| *QSnb.niab-7B.3* | Norway | uLB | 2018 | 7B | 18.74-31.67 | Kukri_c67849_109 and BobWhite_c44404_312 | BS00081132_51 | 3.88 | 5.45 | IM, CIM (cov5, cov10), IBD |

**Table S9** Uncorrected glume blotch severity (uGB) QTL identified in the ‘NIAB Elite MAGIC’ population from field trials conducted in Norway in 2014, 2016, 2017 and 2018, and the UK in 2017 and 2018. QTL with –log_10_(p) value > 3 are presented, with QTL above the permutated trait-specific significance threshold highlighted in bold. Chromosome (Chr.), proportion of the variance explained by QTL (R^2^). Genetic map: Gardner et al. (2016).

| QTL | Location | Trait | Year | Chr | Interval (cM) ^†^ | Flanking markers | Peak Marker | -log_10_ (p) | R^2^ (%) | Detected by QTL methods |
| --- | --- | --- | --- | --- | --- | --- | --- | --- | --- | --- |
| *QSnb.niab-2A.3* | Norway | uGB | 2016 | 2A | 133.18-151.97 | BS00055512_51 and  BS00027830_51 | BS00090569_51 | 3.32 | 3.68 | IM, CIM (cov5, cov10), IBD |
| *QSnb.niab-6A.2* | Norway | uGB | 2016 | 6A | 229.11-238.67 | BS00096240_51 and BS00094893_51 | GENE_4028_152 | 3.23 | 3.77 | IM, CIM (cov5, cov10), IBD |
| ***QSnb.niab-4B*** | **Norway** | **uGB** | **2017** | **4B** | **47.14-57.22** | **BS00100838_51 and BS00076259_51** | **CAP7_c1893_424** | **5.90** | **4.7** | **IM, CIM (cov5, cov10), IBD** |
| ***QSnb.niab-4D*** | **Norway** | **uGB** | **2017** | **4D** | **24.93-34.28** | **Kukri_rep_c68594_530 and RHT2** | **RAC875_c1673_663** | **5.79** | **3.53** | **IM, CIM (cov5, cov10), IBD** |
| *QSnb.niab-5D* | Norway | uGB | 2017 | 5D | 49.43-66.08 | BobWhite_c7263_337 and BS00063971_51 | BS00110475_51 | 3.85 | 3.72 | IM, CIM (cov5, cov10), IBD |

**Table S10** QTL significance -log10(p) thresholds calculated by permutation for each environment and each trait. PH: plant height, DH: Days to heading, uLB: uncorrected leaf blotch disease data, uGB: uncorrected glume blotch disease data

| Environment | Year | PH | DH | uLB | uGB |
| --- | --- | --- | --- | --- | --- |
| Field, Norway | 2014 | 4.28 | 4.24 | 5.34 |  |
| Field, Norway | 2016 | 4.10 | 4.09 | 4.04 | 4.60 |
| Field, Norway | 2017 | 4.13 | 4.58 | 4.28 | 4.30 |
| Field, Norway | 2018 | 4.00 | 4.38 | 4.73 |  |
| Field, UK | 2017 | 4.43 | 4.72 |  |  |
| Field, UK | 2018 | 4.16 | 4.65 |  |  |

|  | **Type** | **Product Name** | **Product Rate** | **Unit** |
| --- | --- | --- | --- | --- |
| ***2017 UK trial*** | |  |  |  |
| 12/09/2016 | Herbicide | Rosate 36 | 0.25 | l/ha |
| 12/09/2016 | Adjuvant | Companion Gold | 2.7 | l/ha |
| 27/10/2016 | Herbicide | Trooper | 4 | l/ha |
| 01/03/2017 | Fertiliser | Origin Sulphur N | 154 | kg/ha |
| 06/04/2017 | Fungicide | Bravo | 1 | l/ha |
| 06/04/2017 | Fungicide | Tebucur | 0.5 | l/ha |
| 13/04/2017 | Fertiliser | Prilled 34.5 N | 217 | kg/ha |
| 28/04/2017 | Herbicide | Starane XL | 1.4 | l/ha |
| 28/04/2017 | Herbicide | Ally Max SX | 35 | g/ha |
| 02/05/2017 | Fungicide | Aviator 235 Pro | 1 | l/ha |
| 02/05/2017 | Fungicide | Bravo | 1 | l/ha |
| 11/05/2017 | Fertiliser | Prilled 34.5 N | 217 | kg/ha |
| ***2018 UK trial*** | |  |  |  |
| 27/10/2017 | Molluscicide | Derrex | 3.5 | kg/ha |
| 14/11/2017 | Herbicide | Avadex | 15 | kg/ha |
| 14/11/2017 | Herbicide | Liberator | 0.6 | l/ha |
| 14/11/2017 | Adjuvant | Backrow | 0.2 | l/ha |
| 16/04/2018 | Fertiliser | Sulphur N | 154 | kg/ha |
| 20/04/2018 | Plant growth regulator | Agrovista 3 See 750 | 1 | l/ha |
| 20/04/2018 | Fungicide | Bravo 500 | 1 | l/ha |
| 20/04/2018 | Fungicide | Tebucur | 0.5 | l/ha |
| 20/04/2018 | Fungicide | Talius | 0.15 | l/ha |
| 21/04/2018 | Fertiliser | Yara prilled 34.5 | 217 | kg/ha |
| 01/05/2018 | Fertiliser | Yara prilled 34.5 | 217 | kg/ha |
| 18/05/2018 | Fungicide | Cherokee | 1 | l/ha |
| 18/05/2018 | Fungicide | Adexar | 1 | l/ha |
| 25/05/2018 | Herbicide | Starane XL | 1.5 | l/ha |
| 25/05/2018 | Herbicide | Ally Max SX | 35 | g/ha |
| 31/05/2018 | Fungicide | Corbel | 1 | l/ha |
| 01/06/2018 | Insecticide | Markate | 0.1 | l/ha |

**Table S11.** Details of the agronomic package used in the UK 2017 and 2018 season trials.

**Supplementary** Text 1

*Five QTL were identified for plant height, on chromosome 2D, 4B, 4D and 6A, respectively. Ten QTL were detected for trait days to heading on chromosome 1B, 2A, 2B, 2D, 3A, 4D, 4D, 5A, 5D, 6A and 6B. For leaf blotch uncorrected data, six QTL were detected on 2A, 3A, 4A, 5B and 7B.*

*We found that ‘strong QTL’ QSnb.niab-6A.1 detected in Norway in 2016 for the corrected leaf blotch phenotype might collocate with one ‘weak QTL’ QDh.niab-6A for days to heading detected in Norway in 2014. However, except that, other colocation of QTL with confounding traits were not found for using both corrected leaf blotch and uncorrected leaf blotch phenotypes.* ‘Strong QTL’ *detected with uncorrected disease data all had been detected by previous corrected disease data (QSnb.niab-2A.3 and QSnb.niab-3A). In total, five ‘weak QTL’ were detected by uncorrected leaf blotch data. QSnb.niab-2A.3 and QSnb.niab-2A.4 had been detected previously using corrected leaf blotch phenotypes, but three other ‘weak QTL’ were not detected by corrected disease data.*

*For glume blotch, five QTL were detected with the uncorrected data, however all Strong QTL collocated with the plant height QTL on chromosome 4B and 4D. The rest three glume blotch QTL were previously identified by corrected disease data, however mostly with less significance using uncorrected disease data.*
