# Supplementary figures and images for: Genetic mapping using a wheat multi-founder population reveals a locus on chromosome 2A controlling resistance to both leaf and glume blotch caused by the necrotrophic fungal pathogen Parastagonospora nodorum
Source: Theor Appl Genet. 2020 Jan 29;133(3):785–808. doi: 10.1007/s00122-019-03507-w (PMC7021668; doi:10.1007/s00122-019-03507-w)

5B

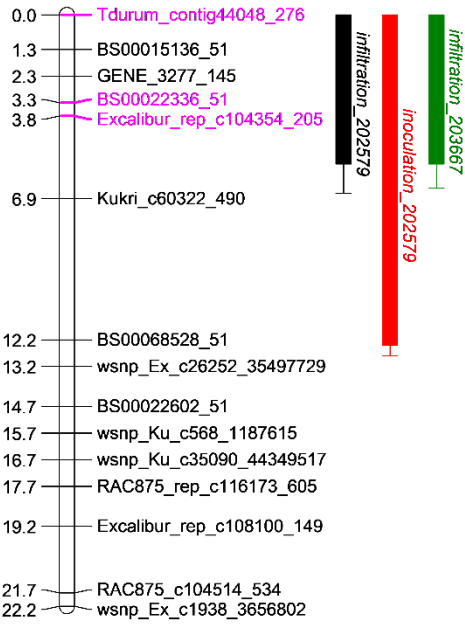

Supplement: Supplementary file 2 — Genetic map of the Snn3-B1 locus detected by greenhouse experiment on the short arm of chromosome 5B (QSnb.niab-5B.1) in the NIAB Elite MAGIC population. Peak markers are indicated in pink. (PDF 75 kb) [file 122_2019_3507_MOESM2_ESM.pdf]

5B

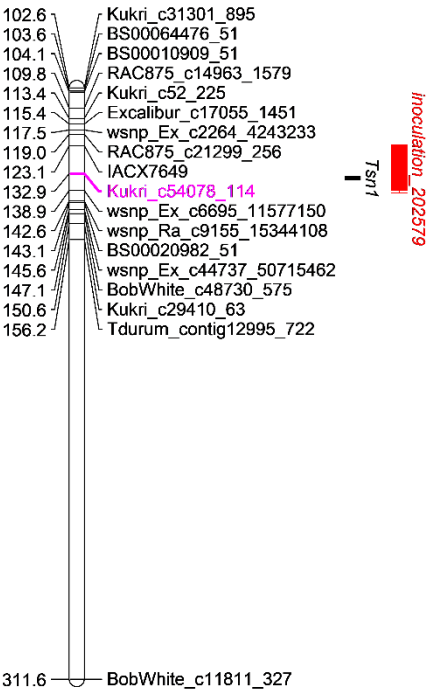

Supplement: Supplementary file 3 — Genetic map of the Tsn1 locus detected by seedling inoculation using isolate 202579 on the long arm of chromosome 5B (QSnb.niab-5B.2) in the ‘NIAB Elite MAGIC’ population. Peak markers are indicated in pink. (PDF 78 kb) [file 122_2019_3507_MOESM3_ESM.pdf]

7D

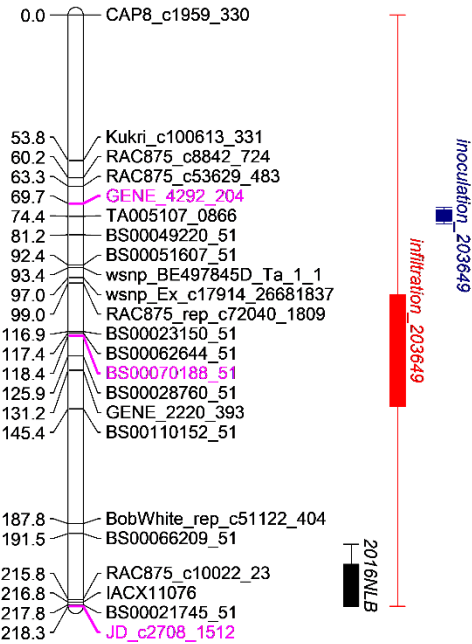

Supplement: Supplementary file 4 — Genetic map of the QSnb.niab-7D.1 and QSnb.niab-7D.2 loci, detected by infiltration of isolate 203649, and QSnb.niab-7D.3 detected by leaf blotch 2016 in Norway on chromosome 7D in the ‘NIAB Elite MAGIC’ population. N: Norway, LB: leaf blotch, Peak markers are indicated in pink. (PDF 92 kb) [file 122_2019_3507_MOESM4_ESM.pdf]

3A

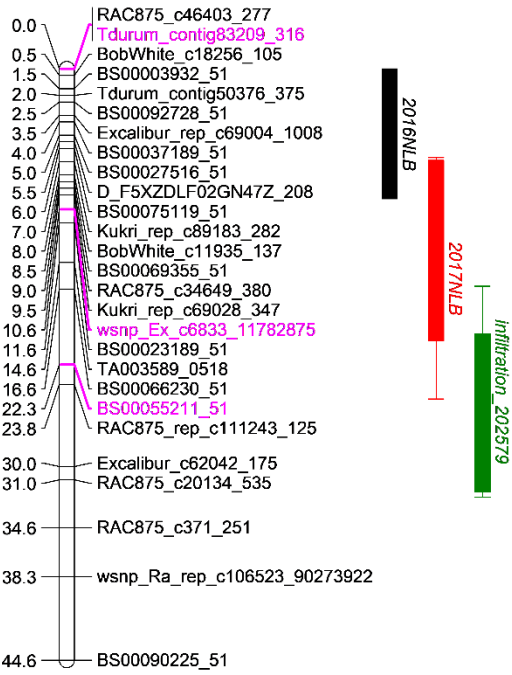

Supplement: Supplementary file 5 — Genetic map of the QSnb.niab-3A locus on the short arm of chromosome 3A in the ‘NIAB Elite MAGIC’ population. N: Norway, LB: leaf blotch. Peak markers are indicated in pink. (PDF 106 kb) [file 122_2019_3507_MOESM5_ESM.pdf]

6A

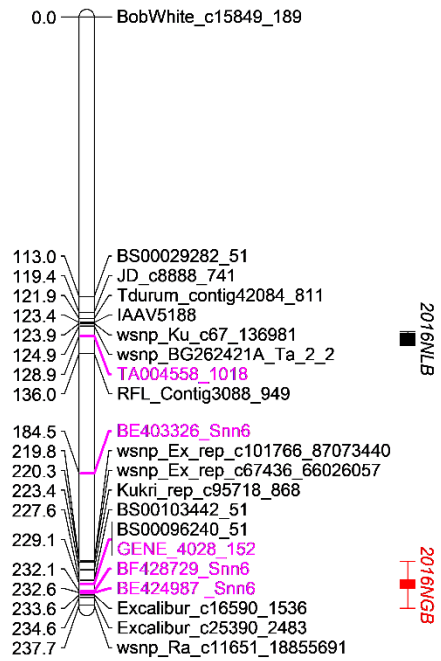

Supplement: Supplementary file 6 — Genetic map of the QSnb.niab-6A.1 and QSnb.niab-6A.2 loci, detected by glume blotch 2016 in Norway on chromosome 6A in the ‘NIAB Elite MAGIC’ population. N: Norway, LB: leaf blotch, GB: glume blotch. Peak markers and Snn6 markers (Gao et al. 2015) are indicated in pink. (PDF 87 kb) [file 122_2019_3507_MOESM6_ESM.pdf]

a

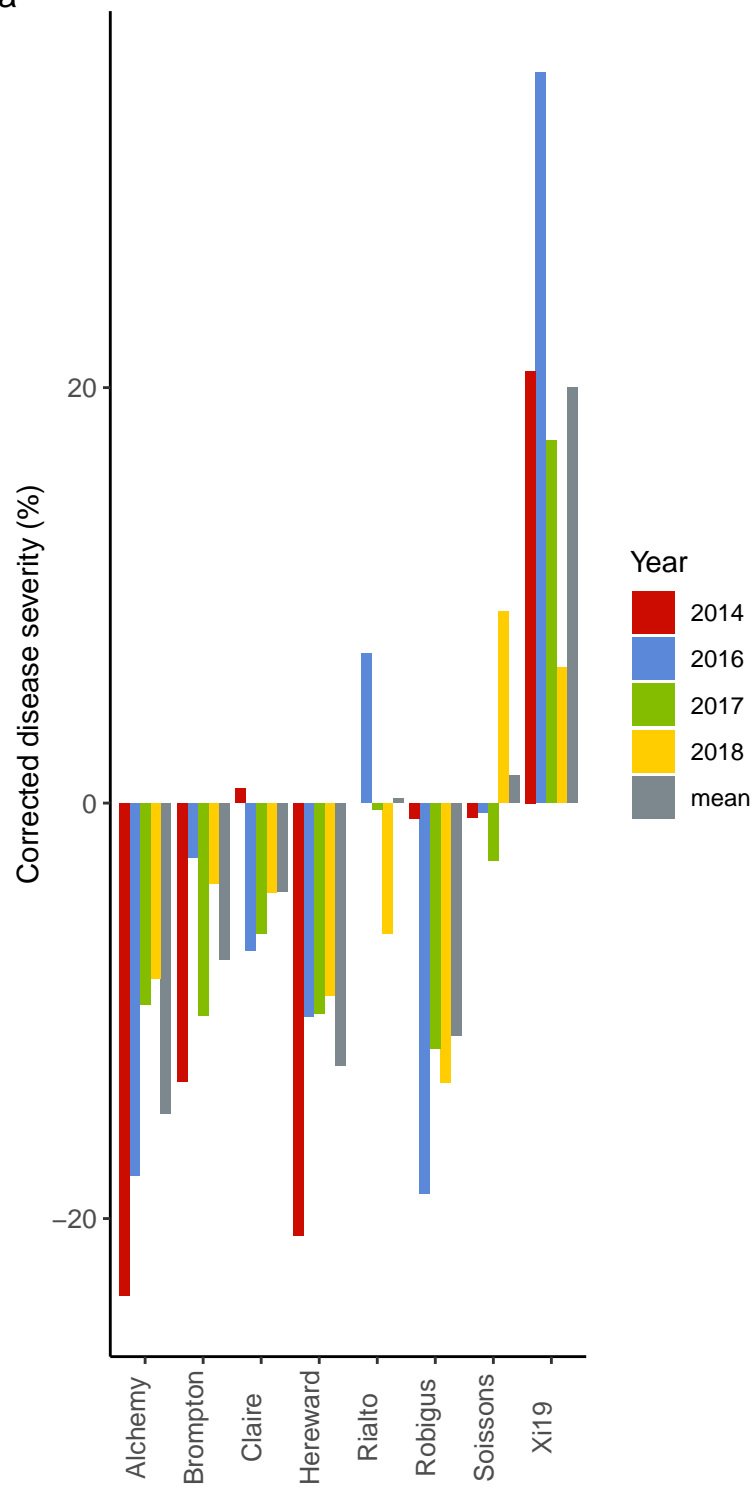

b

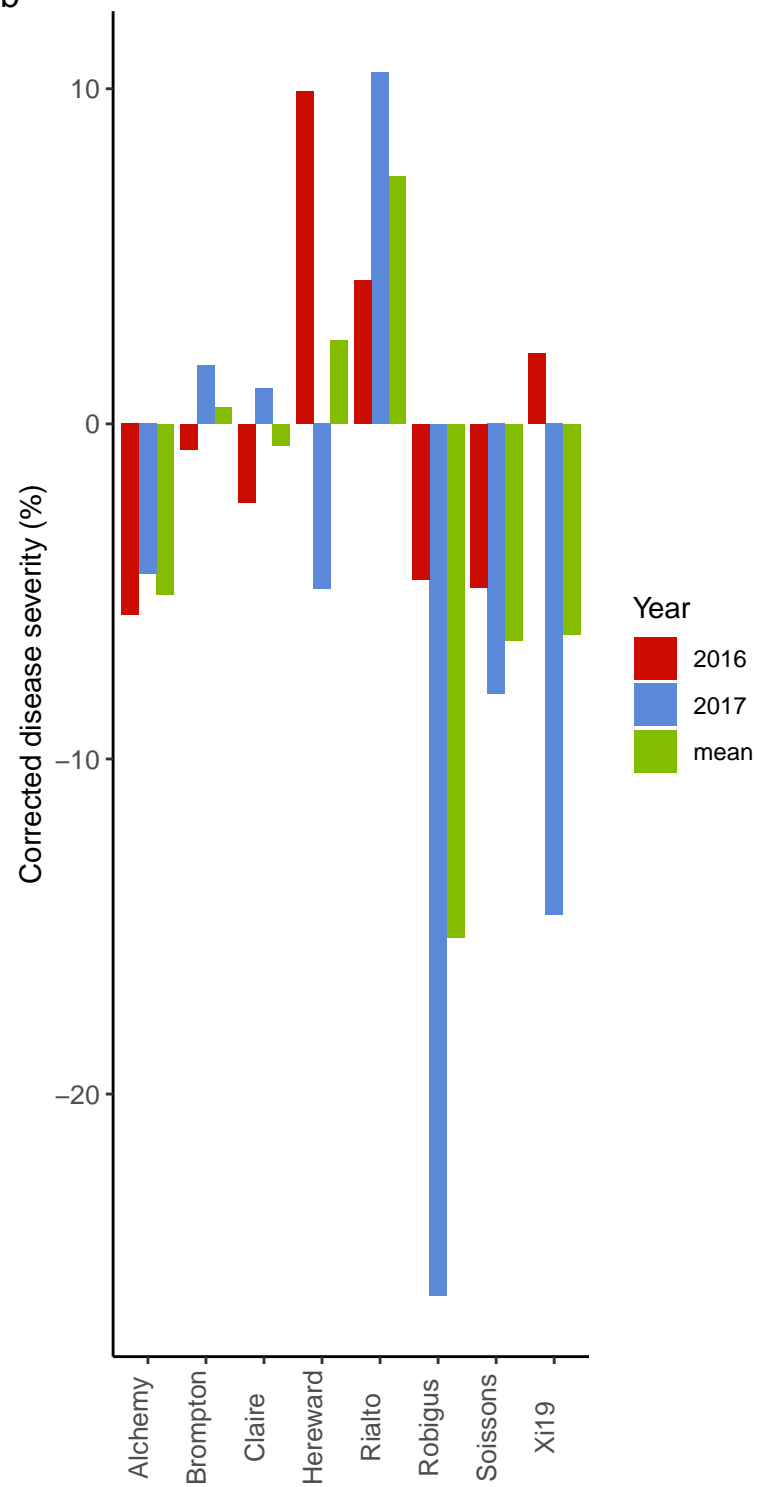

Supplement: Supplementary file 7 — Corrected disease severity of MAGIC founders in different years and locations. (a) Leaf blotch severity in Ås, Norway, (b) glume blotch severity in Ås, Norway. (PDF 5 kb) [file 122_2019_3507_MOESM7_ESM.pdf]
